# EZS-SEP: A hybrid efficient zonal stable election routing protocol with SOM-based node deployment for heterogeneous wireless sensor networks

**DOI:** 10.1371/journal.pone.0346479

**Published:** 2026-08-27

**Authors:** Sumit Das, Md. Khorshed Alom

**Affiliations:** Department of Electronics and Communication Engineering, Khulna University of Engineering & Technology, Khulna, Bangladesh; Anurag University, INDIA

## Abstract

Wireless Sensor Networks (WSNs) are widely utilized in numerous applications for data collection and monitoring; however, their performance is often limited by energy constraints and the need for efficient data transmission. Network stability largely depends on node energy utilization, clustering strategy, and data transmission methods. In this work, an energy-efficient routing protocol is introduced for heterogeneous WSNs, referred to as Efficient Zonal Stable Election Routing Protocol with SOM-based node deployment (EZS-SEP). The proposed protocol aims to enhance network stability, area coverage, throughput, and overall performance by employing a hybrid communication technique and a self-organizing map (SOM) for node deployment. The WSN is divided into distinct zones based on energy levels. Normal nodes with lower energy are placed near the Base Station (BS), while intermediate and advanced nodes are positioned farther from the BS. This arrangement ensures optimal energy utilization. The Cluster Head (CH) selection mechanism of the protocol considers several critical factors, including residual energy, distance from the BS, and average node energy, ensuring that higher-energy nodes with favorable proximity are elected as CHs. Simulation results demonstrate that EZS-SEP outperforms conventional heterogeneous clustering protocols in terms of stability period, network lifetime, and throughput. Therefore, for real-world WSN applications requiring energy efficiency and reliable coverage, EZS-SEP is a suitable solution. It improves stability by 34%, 63%, 31%, and 174% compared to ESEP, P-SEP, Z-SEP, and LEACH, respectively. This study further evaluates the performance of EZS-SEP under different scenarios, including variations in BS position and initial energy levels, demonstrating its robustness across diverse network conditions.

## 1. Introduction

Wireless Sensor Networks (WSNs) have attracted increasing attention owing to their wide applications including environmental monitoring, agricultural management, health monitoring, industrial automation, smart homes and cities, as well as disaster search and rescue [1,2]. This popularity has brought about groundbreaking progress in the field of Internet of Things (IoT), Internet of Everything (IoE) and Vehicular Ad Hoc Networks (VANET), particularly in terms of compact size, low deployment cost and user-friendly interfaces [3]. WSNs consist of numerous small, randomly or regularly distributed sensor nodes designed to monitor environmental factors such as temperature, humidity, pressure, sound, motion and vibration. These sensors are small battery-powered devices equipped with small processors and confined storage and radio capabilities. Reference [4] outlines that a sensor node operates through three main subsystems: the sensing subsystem, which detects environmental conditions; the processing subsystem, which performs local data computation; and the communication subsystem, which handles data exchange with nearby nodes. In addition to capturing real-time data from their environment to monitor and evaluate different physical parameters, these sensors also process and transmit aggregated information to users with a reliable quality of service [5]. In WSNs, after collecting the data from the surroundings, the sensor nodes report to the Base Station (BS) commonly known as “sink”. A Sink Node (SN) refers to a node equipped with greater processing capabilities, larger storage capacity and a rechargeable power source [6]. The data can be forwarded to sink from the nodes via direct transmission technique and clustering approaches or transmitting through cluster-heads (CHs) [1,7].

Since these nodes rely on non-rechargeable batteries, their energy gradually depletes as they continuously transmit, receive and process data. Energy consumption has a significant impact on the performance of WSNs. Given that each sensor is powered by a limited battery capacity, frequent data transmission and processing can quickly drain its power. As nodes begin to fail, the network loses coverage and data routing becomes less reliable. Therefore, optimally assigning sensors to tasks and utilizing energy efficiently is crucial to prolong the lifespan of WSNs [8]. Ensuring energy efficiency is essential not only to maintain stable connectivity and accurate data collection, but also to reduce maintenance costs and extend the network’s overall lifespan. Direct Transmission (DT) [9] and Minimum Transmission Energy (MTE) [10] are classical approaches that fail to distribute energy consumption evenly across sensor nodes. In DT, distant nodes drain their energy quickly by sending data directly to the sink. In MTE, nearby nodes are overused as relays, causing them to die early due to heavier energy load. These traditional techniques cannot provide full coverage of the field as a result; the collected data becomes unreliable. To address this problem, designing an optimal routing protocol is crucial, leading to the development of numerous energy-efficient routing techniques in recent years [11].

To manage data collection and transfer to the BS, various routing protocols have been proposed, but their design faces several challenges, such as node deployment, sensor positioning, transmission range and network lifetime, with energy consumption being the most critical concern. The deployment of nodes in a WSN generally falls into two categories: (1) structured and (2) unstructured. In unstructured WSNs, sensor nodes are randomly scattered across the target field. Although this method allows for quick and large-scale deployment, it is important to develop strategies to improve network management, particularly for failure detection and connectivity maintenance. Structured WSNs, on the other hand, involve devices being systematically placed in a well-organized and predetermined manner, making them easier to manage and monitor [12]. Furthermore, sensor nodes may be deployed in either a (1) controlled or (2) uncontrolled environment [13]. In a controlled environment, the positions of all nodes can be monitored and the sensors are easily accessible and routinely maintained. On the other hand, in an uncontrolled environment, sensor nodes are deployed in inaccessible areas, where there is no fixed deployment pattern and they are not regularly serviced. The configuration of a WSN typically consists of a BS and multiple sensors distributed across a region. Each node can detect environmental events, analyze them and then forward the data to the BS either in a multi-hop fashion or via a direct link [13]. The sensors monitor the area, collect data, process it and transmit it to the BS. When messages are sent directly to the BS, sensor nodes require a considerable amount of power to relay data, causing their energy to deplete rapidly. In contrast, during multi-hop transmission, data is forwarded through several intermediate nodes before reaching the BS, thereby reducing overall energy consumption.

A basic classification of sensor networks based on their operating mode and the nature of the target application can be divided into: (1) Proactive Routing Protocols and (2) Reactive Routing Protocols [14,15]. In proactive protocols, nodes in the network continuously sense and report data by regularly monitoring their surroundings, activating their transmitters and sending information periodically. This makes them suitable for applications requiring consistent, real-time updates. In contrast, reactive protocols involve nodes that continuously monitor the environment but transmit data only when a significant change in the sensed values occurs. Therefore, time-sensitive applications are a good fit for reactive networks.

Since WSNs have limited energy resources, various routing strategies have been proposed for both homogeneous and heterogeneous networks. In homogeneous WSNs, all sensor nodes are identical in terms of energy capacity, communication range and computational capability. Heterogeneous WSNs, on the other hand, consist of nodes that differ in design, energy level and processing power. These networks are more practical for real-world deployment, as they offer better performance and flexibility with minimal impact on overall deployment cost. This paradigm shift has led to increased research interest in heterogeneous WSNs over their homogeneous counterparts, due to their enhanced adaptability and higher applicability in real-world scenarios [17].

**Fig 1:**
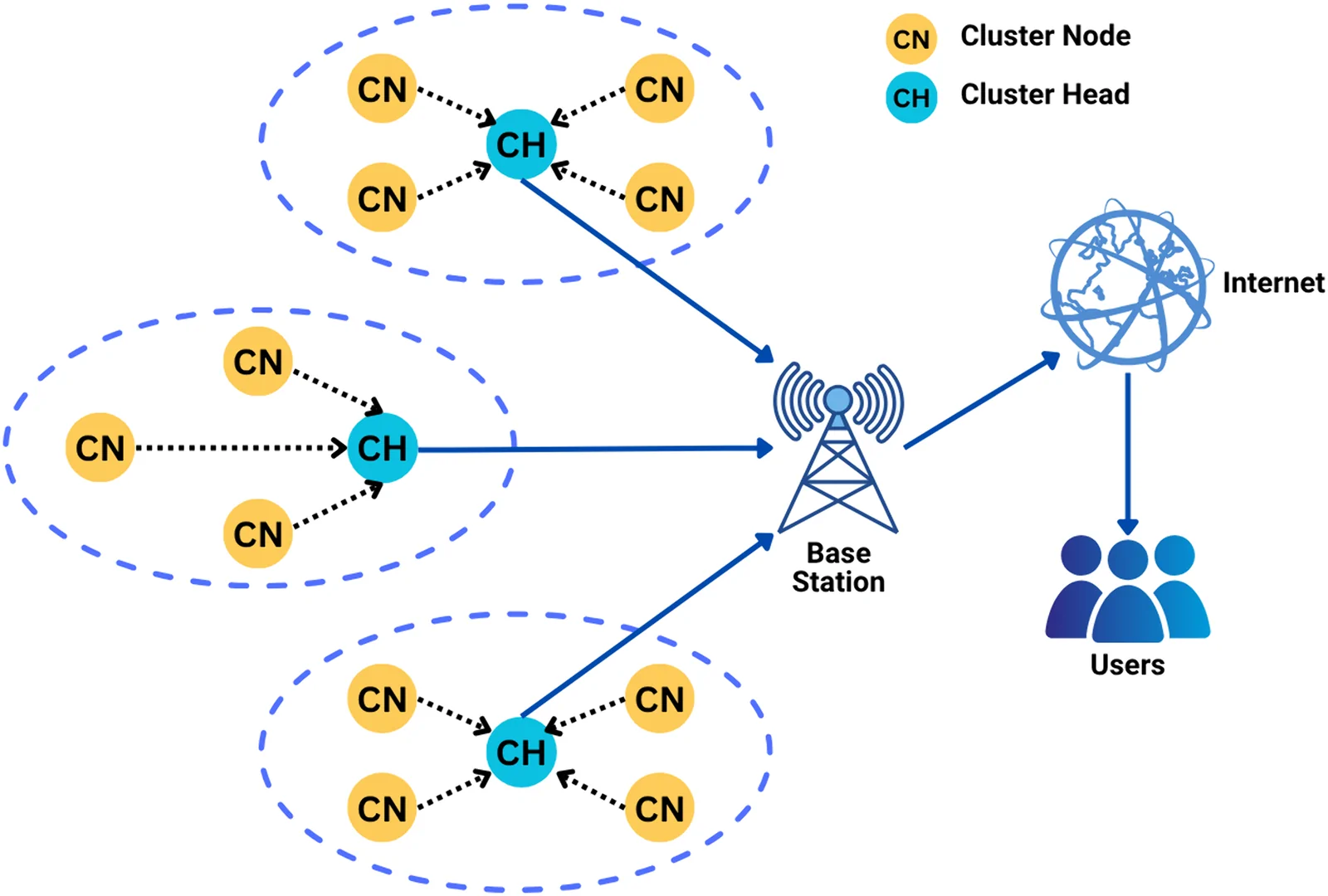
Cluster-based WSN architecture.

Energy efficiency is considered the primary concern in WSNs. Clustering helps reduce the energy usage of sensor nodes. Numerous energy-efficient techniques have been developed with an emphasis on clustering [9,16,17]. In clustering, nodes are organized into clusters, where only one node acts as CH. Each node that belongs to a cluster transmits its sensed data to the CH and the CH aggregates the received data before relaying it to BS. The process of clustering is useful in reducing communication overheads, thereby minimizing the overall energy usage of the network. Hence, the selection of CHs should be performed wisely to create a balanced network with minimal use of available energy. Fig 1 illustrates a cluster-based WSN architecture. In a homogeneous network, each node in the cluster has an equal chance of becoming a CH. After selection, the CH carries out data aggregation and sends it to the BS, which results in a heavy communication load. Therefore, the CH expires first, followed by other nodes, resulting in network instability [13]. In heterogeneous networks, nodes have different energy levels and varying CH selection probabilities [17]. Network performance becomes fragile when one of the nodes fails. To this end, various routing protocols have been proposed to enhance stability duration and extend the lifetime of the sensor network. A notable cluster-based hierarchical protocol in WSNs is the Low Energy Adaptive Clustering Hierarchy (LEACH) protocol [9]. Nevertheless, LEACH is applicable only to homogeneous WSNs and since CHs are randomly selected, the protocol fails to guarantee optimal CH selection. To prolong network stability and preserve node heterogeneity with optimal CH selection, the Stable Election Protocol (SEP) [17] and the Distributed Energy-Efficient Clustering (DEEC) algorithm [16] have been proposed. Many derived protocols have been developed based on these protocols which have significantly improved the network lifetime.

Together with such advancements, there is also a significant concern regarding network field coverage. The issue of providing adequate sensing coverage is one of the critical challenges that has received substantial attention in recent years [18], particularly in the context of achieving maximum sensing coverage across the Region of Interest (ROI). This required coverage can be achieved through the precise placement of sensor nodes following the initial deployment. As such, random placement of mobile sensors cannot guarantee proper coverage within the ROI. For sensor deployment, different algorithms with varied approaches have been developed to enhance communication among neighboring nodes and improve the overall coverage range of the monitored area [18,19]. In the event of static node deployment, self-organizing maps [20,21] and hebbian machine learning [22] can be utilized. In the case of static sensors, complete coverage is essential, as any omission in sensor placement leads to direct blind spots in the network. These partial coverage gaps significantly affect the accuracy and quality of the collected data, thereby resulting in erroneous or incomplete environmental analysis. Moreover, the loss of data reliability caused by insufficient coverage becomes even more critical in the context of sensitive applications, such as disaster monitoring or industrial control systems that involve highly critical operations. In such cases, inadequate coverage may cause severe misinterpretation and lead to potentially disastrous outcomes.

### 1.1. Motivation and Issues in the Existing System

Z-SEP protocol [23] is a heterogeneous clustering-based routing protocol for WSNs, designed for networks where nodes possess different initial energy levels. In Z-SEP, nodes employ a hybrid communication scheme with the BS that combines direct transmission and CH-based communication. There are two main categories of nodes: (1) normal nodes, which have comparatively lower initial energy and are typically deployed closer to the BS and (2) advanced nodes, which have higher energy levels and are deployed farther from the BS. The sensing field is divided into three zones to facilitate efficient sensor deployment and communication management. Normally, nodes located in the first zone nearest to the BS are normal nodes, whereas advanced nodes are uniformly distributed across the two outer zones. The normal nodes communicate directly with the BS, while the advanced nodes form clusters, where CHs aggregate data from member nodes and transmit it to the BS. Nevertheless, several drawbacks emerge in realistic large-scale scenarios, particularly when the network field size increases or node mobility is introduced. As the field expands, advanced nodes located farther from the BS experience higher energy consumption due to the increased transmission distance, leading to rapid battery depletion. Consequently, the overall network lifetime decreases, and CH selection among advanced nodes becomes unstable. Moreover, there are some intrinsic issues that worsen the network stability:

- The direct communication between sensor nodes and the BS consumes a significant amount of energy. Since sensor nodes have limited battery capacity and computational resources, this approach subjects the advanced nodes functioning as CHs to rapid energy depletion, as they are continuously engaged in data collection, aggregation and transmission. This accelerates their energy exhaustion, causing network instability and reducing the overall lifespan of the WSN.
- This instability is further enhanced by the random CH election process among advanced nodes, which can result in the selection of low-energy nodes as CHs. Such nodes quickly deplete their remaining energy, lose CH functionality prematurely and trigger cluster disconnections, leading to network instability and degraded performance, especially when multiple nodes begin to fail simultaneously.
- As the network field dimensions increase, both normal nodes (direct communicators) and advanced nodes (serving as CHs) experience higher energy consumption due to the greater internal transmission distances between nodes and the BS. Since energy consumption is directly proportional to the transmission distance, the network becomes unstable and the negative effects extend to reliability, coverage efficiency and overall performance of the Z-SEP routing protocol.
- Finally, when node deployment fails to cover the entire sensing field, the collected data becomes incomplete and unreliable, creating blind spots that compromise monitoring accuracy. These blind spots further degrade decision-making quality and reduce the overall efficiency of the WSN.

### 1.2. List of Major Contributions

In this paper, a proactive routing protocol is introduced that considers a heterogeneous setting with three different energy levels. The main differences between the parent Z-SEP [23] protocol and our proposed protocol is the consideration of different energy and distance parameters in the probability and threshold equation along with adopting three-level heterogeneity and utilizing specialized deployment technique. We assumed the field is divided into several zones, where lower-energy nodes are positioned closer to the BS, while higher-energy nodes are placed farther away. The nodes are distributed using a self-organizing technique for better coverage. Based on these assumptions, we introduce a new protocol called Efficient Zonal Stable Election Routing Protocol with SOM-based node deployment (EZS-SEP). In the EZS-SEP protocol, advanced and intermediate nodes are selected as CHs that transmit data to the BS, whereas normal nodes communicate with the BS directly. The main contributions of this research are outlined as follows:

i. Proposes a routing protocol which employs a multi-factor CH selection strategy based on residual node energy, initial node energy, node distance to the BS, average distance of all alive nodes from the BS, and average residual energy of all alive nodes. By favoring high-energy nodes closer to the BS, it reduces transmission energy consumption, balances energy usage, and prolongs network stability by delaying the first node death.
ii. The proposed protocol incorporates a three-level heterogeneous energy model to improve the overall energy efficiency of the network.
iii. Adopts a Self-Organizing Map (SOM)-based node deployment strategy to optimize zonal network coverage and enhance data reliability.
iv. Provides comprehensive simulation results which demonstrate the proposed protocol outperforms existing baseline routing protocols in terms of stability period, network lifetime, throughput, and energy efficiency.
v. The effectiveness of the proposed protocol will be assessed across multiple network scenarios: (1) Changing the initial energy of nodes (2) Changing the position of the BS (3) Changing the network size.

### 1.3. Paper Organization

The rest of the paper is structured as follows: Section 2 provides a brief overview of related research. Section 3 describes the system models used in this study. Section 4 presents the proposed energy-efficient WSN routing protocol. Section 5 discusses the evaluation metrics and presents the simulation results along with a comparison against existing protocols. Finally, Section 6 concludes the paper and outlines possible avenues for future research.

## 2. Related Works

In recent years, energy efficiency, clustering, routing and coverage have become critical issues in WSNs, as these factors significantly influence the network’s overall performance. The primary role of sensor nodes is to sense their environment and collect the relevant data, perform limited local processing and aggregation and send this data to the BS. Although direct interaction between the BS and the sensor nodes is a straightforward approach, it rapidly drains the nodes, leaving them without enough energy to complete the mission and hence causing early node failures. As a result, protocols that improve energy efficiency by transmitting information via several nodes have emerged as a focus of research. The principal aim of such protocols is the minimization of energy consumption to extend the operational lifetime and enhance the reliability of WSNs.

LEACH [9] is the pioneer hierarchical routing protocol for homogeneous WSNs designed to enhance network lifetime. LEACH uses a randomized rotation scheme that uniformly distributes the energy load among sensor nodes in the network. The LEACH process consists of two phases: (1) Setup phase and (2) Steady-state phase. During the setup phase, clusters are formed and CHs are selected. A pre-defined node probability "p" determines whether a node identifies itself as a CH in the following way: the node chooses a random number between 0 and 1. If the random number is less than the threshold valueT(n), then it is chosen as the CH for the current round. The threshold is calculated by the following formula:


$$\begin{array}{c} T(n)=\left\{ \begin{array}{c} @l\frac{p}{\text{1}-p(rmod\frac{\text{1}}{p})},\;ifn\in G\\ \text{0},\;otherwise \end{array}\right. \end{array}$$ (1)

Where, r indicates the current round and G is a set of nodes that have not been selected as CHs in the last $\frac{\text{1}}{p}$ rounds. In every $\frac{\text{1}}{p}$ round, each node gets the chance to elect as CH exactly once. So, all nodes have the same selection probability and the same initial energy denoted as $E_{node}=E_{o}$. The total energy of the network can therefore be expressed as $E_{Total}={nE}_{o}$.

Nevertheless, the original LEACH does not perform well even under optimal energy distribution, particularly in large-scale WSNs; therefore, numerous enhanced LEACH-based protocols have been proposed. Several sophisticated extensions of LEACH include LEACH-DCHS [24], LEACH-DT [25], Improved LEACH [26], HEED [27] and ALEACH [28]. In LEACH with Deterministic Cluster Head Selection (LEACH-DCHS), the criterion for determining which node becomes a CH is computed based on the residual energy level of each node [24]. Subsequently, LEACH with Distance-based Threshold (LEACH-DT) incorporates the sensor to BS distance into the CH election mechanism [25]. Furthermore, Improved LEACH integrates both energy and distance factors into the threshold function, combined with a multi-hop routing algorithm that utilizes hop-count and residual energy for next-hop selection [26]. The HEED protocol builds upon the fundamental principles of the LEACH protocol and considers both the residual energy of each sensor node and the intra-node distance to its neighbors during CH selection; however, it does not account for sensor node density [27]. The Advanced LEACH (ALEACH) protocol introduces heterogeneity into LEACH and rotates CH positions to distribute energy consumption more uniformly across all nodes [28]. Meenakshi et al. [29] proposed an optimized energy-efficient Engroove LEACH clustering protocol that combines Modified Improved Harris Hawks Fitness Optimization (MIHFO) for CH selection and Hybrid Whale Archimedes Optimization (HWAO) for route optimization. The protocol improves energy efficiency, and network lifetime compared with LEACH, and DEEC by jointly optimizing clustering and routing. Another homogeneous routing protocol worth mentioning is the Power-Efficient Gathering in Sensor Information Systems (PEGASIS) [30], a chain-based protocol that minimizes energy consumption by enabling nodes to transmit data through their immediate neighbors. Improved variants of PEGASIS include IEEPB [31] and MIEEPB [32].

Since low power consumption and prolonged operational lifetime are the foremost criteria in WSN design, researchers have investigated node-level energy heterogeneity to enhance network stability. Subsequently, Smaragdakis et al. [17] proposed the SEP, which is explicitly defined for heterogeneous networks comprising two categories of nodes: advanced nodes, which possess higher initial energy and normal nodes, whose initial energy is lower. The initial energy for normal nodes is $E_{o}$, while the initial energy for advanced nodes is $E_{adv}=E_{o}(\text{1}+\alpha )$, where α denotes the additional energy factor distinguishing advanced nodes from normal nodes. If m represents the fraction of advanced nodes in the network, then the total network energy increases to:


$$\begin{array}{c} E_{Total}=n\times (\text{1}-m)\times Eo+n\times m\times Eo(\text{1}+\alpha )=n\times Eo\times (\text{1}+\alpha m) \end{array}$$ (2)

SEP stabilizes the network by scaling the probability of CH selection in accordance with the nodes’ initial energy. The nodes that have greater initial energy, which are advanced nodes, get the greater probability to become CHs. To ensure fairer energy distribution, the CH election probabilities are therefore energy-weighted, defined as:


$$\begin{array}{c} P_{nrm}=\frac{P_{opt}}{\left(\text{1}+\alpha m\right)} \end{array}$$ (3)

$$\begin{array}{c} P_{adv}=\frac{P_{opt}*(\text{1}+\alpha )}{\left(\text{1}+\alpha m\right)} \end{array}$$ (4)

Similar to SEP, another heterogeneous protocol was proposed, known as the DEEC protocol [16]. DEEC enhances network scalability and prolongs network lifetime by selecting CHs based on the residual energy of each node relative to the average energy of the network. Variants of DEEC protocol have considered different conditions to improve the performance of the routing protocol. Jibreel et al. [33] proposed Distance-Distributed Energy Efficient Clustering (D-DEEC), an enhanced heterogeneous routing protocol that incorporates individual node distance and average distance from the BS alongside residual energy in CH election, further employing a sleep and awake mechanism for distant nodes. However, SEP cannot efficiently exploit the excess energy of advanced nodes because it does not incorporate residual energy into CH selection. Moreover, SEP does not ensure effective node distribution. Consequently, several modifications of SEP have been introduced. The Distance-Based SEP (DB-SEP) [34] was proposed to select CHs using both node energy and sink distance, while employing multi-hop communication and data aggregation to reduce overall transmissions. HSEP [35] is a hierarchical routing protocol designed for heterogeneous WSNs with the objective of improving energy utilization. To minimize the transmission distance to the BS, it introduces two layers of CHs: primary and secondary cluster heads. Only high-energy nodes have a higher likelihood of becoming CHs; this depends on their initial energy and a predefined probability threshold. Primary CHs collect information from their member nodes and forward it to the nearest CH designated as a secondary CH, which then relays the aggregated data to the BS. This two-tier communication scheme reduces overall power consumption and enhances network sustainability. Assuming multi-level heterogeneity and two aggregator nodes that forward data from CHs to the sink through the nearest aggregator, the Modified SEP (M-SEP) [36] reduces transmission energy. Nevertheless, the proposed approach cannot effectively prolong network lifetime under all conditions. In [37], EM-SEP is introduced, a refined version of SEP that selects advanced nodes more frequently as CHs, accounts for cluster size and chooses the highest-energy node when multiple candidates are available, thereby improving energy equilibrium and extending network lifetime. In [6], the authors proposed a composite solution called Distance-Aware Residual Energy-Efficient SEP (DARE-SEP) for both homogeneous and heterogeneous WSNs. In DARE-SEP, CHs are selected based on the node’s residual energy, its distance from the BS and weighted factors that prioritize nodes with higher energy and those located closer to the BS. Nonetheless, an effective selection of these weight values is crucial for system performance, yet this aspect is not thoroughly examined in the presented results. For a heterogeneous setting, the threshold is given as:


$$\begin{array}{c} T(n)=\left[w\text{1}\left(\frac{E_{n}}{E_{max}}\right)+w\text{2}\left(\frac{D_{avg}}{D_{bs}}\right)\right]\times \left[\frac{P(n)}{\text{1}-P(n)(r\times mod(\frac{\text{1}}{P(n)}))}\right] \end{array}$$ (5)

Where w1 and w2 are the network’s energy and distance priority weights, which are equal to one. A significant improvement of the SEP protocol is observed in the Distance and Energy Aware SEP (DE-SEP) [38]. In DE-SEP, the weighted probabilities for three-level heterogeneous nodes are computed using the initial energy and the average energy of the alive nodes. Based on each node’s distance from the BS and the average distance between all alive nodes and the BS, the threshold equation incorporates two cases, where residual energy is also accounted for in determining the CH selection threshold [38]. Our proposed protocol employs an even advanced energy factor than DE-SEP to prolong the network lifetime and also uses a specific zonal deployment structure for hybrid communication. DE-SEP’s dependance on CH based communication may limit its scalability and lifetime. Prolong-SEP (P-SEP) [39] was proposed as an enhanced version of SEP for fog-supported WSNs. The P-SEP protocol strengthens CH selection in a two-level fog-based WSN by exploiting energy differences, reducing repetitive CH elections and equalizing the load through energy-aware decisions and optimal CH–FN distance selection. An Enhanced Stable Election Protocol [40] considered three levels of heterogeneity while keeping the standard SEP probability and threshold equations unchanged. Some methods adopted a super-heterogeneous configuration, such as BEENISH [41], which used four levels of nodes within the network. In [42], the authors proposed a SEP-based routing protocol for HWSNs that used TDoA (Time Difference of Arrival) to form energy-efficient clusters by minimizing node-to-CH distance. While most protocols considered a two-dimensional architecture, Baghouri et al. [43] generalized the classic 2D SEP to 3D sensor deployments, analytically deriving optimal cluster counts and CH-selection probabilities using x, y, z distance models and adapting SEP’s heterogeneity thresholds accordingly. A four-level heterogeneous clustering protocol, MCZSEP-HN is proposed in [44] that combines circular zone-based deployment with helper nodes to reduce transmission distance and improve energy distribution. The protocol relies on fixed helper-node placement and static zone partitioning, which limits its adaptability to dynamic or randomly deployed WSNs. An energy-efficient Knapsack-based CH selection algorithm [45] have been proposed that formulates CH selection as a 0/1 Knapsack optimization problem, where candidate CHs are evaluated according to their residual energy, communication overhead, and proximity to the BS, and the nodes with the highest value-to-weight ratio are selected to achieve balanced energy consumption and efficient cluster formation. In [46], Quantum Particle Swarm Optimization (QPSO) is employed to determine the optimal CHs by considering residual energy and distance to the BS. It then applies a fuzzy logic system to select relay nodes using residual energy, energy deviation, and relay distance, enabling energy-balanced multi-hop routing. Another study presented a neuro-fuzzy data routing (NFDR) [47] mechanism that combines neural networks with fuzzy logic to optimize CH selection and routing decisions, enhancing data transmission performance in IoT-enabled WSNs. Kumar et al. [48] proposed EEECA-THWS, which employs a modified threshold-based cluster-head selection using residual energy and distance to balance energy consumption among three-tier heterogeneous sensor nodes. It relies on single-hop communication between CHs and the BS, making it less energy-efficient for large-scale WSNs.

Continuous data transmission is not required in many WSN applications and therefore falls under reactive routing protocols. In reactive protocols, data communication is triggered only when the sensed information exhibits a significant change. Threshold Sensitive SEP (TSEP) [15] is a reactive protocol that incorporates three-level node heterogeneity. This protocol introduces Hard Threshold (HT) and Soft Threshold (ST) parameters to regulate data transmission. However, TSEP does not ensure a balanced distribution of network load. Enhanced Threshold Sensitive SEP (ETSSEP) [49] extends TSEP by incorporating the residual energy of nodes into the threshold formulation and by considering the minimum number of clusters per round in its CH selection strategy. Further modification on ETSSEP was proposed in DETSSEP [50]. In DETSSEP, the threshold computation additionally considers remaining energy, average network energy, node-to-BS distance and the number of rounds since the node last served as a CH. This protocol also employs single-hop and dual-hop communication, contributing to an improvement in overall network lifetime.

In many protocols, hybrid data communication and special zonal distribution of nodes are employed. Normal nodes consume more energy during data transmission when they are positioned farther from the BS, which increases overall energy usage, leading to reduced stability and lower throughput. To overcome these drawbacks, the concept of network field partitioning combined with a hybrid communication method has been introduced. In Z-SEP [23], the network is divided into three zones: Zone 0 contains normal nodes located near the BS, while Zones 1 and 2 contain advanced nodes placed farther from the BS. Normal nodes communicate directly with the BS through direct communication, whereas advanced nodes communicate via clustering. The Z-SEP threshold equation follows the standard LEACH [9] protocol, and the weighted probability for advanced nodes follows the standard SEP [17] formulation. To maintain the stability period, clusters are formed only by high-energy advanced nodes located farther from the BS, while normal nodes near the BS transmit data directly to the BS. Since CHs are selected based on a constant threshold and randomly from all advanced nodes, low-energy sensors may be chosen, causing them to deplete their batteries rapidly, destabilizing clusters upon their failure and potentially leaving clusters without a CH. Modifying the CH selection criteria to incorporate residual energy along with the distance to the BS can prevent the premature failure of CHs and ensure more efficient energy distribution. To address this issue, the authors in [13] introduced AZ-SEP, which incorporates both residual energy and distance factors for CH selection. The factor is calculated as:


$$\begin{array}{c} P(n)=\frac{E_{res}(n)}{E_{avg}}*\frac{D_{max}}{D_{bs}} \end{array}$$ (6)

Despite considering key parameters in CH selection, AZ-SEP fails to account for network coverage and advanced energy heterogeneity. In contrast, our proposed protocol employs self-organizing maps to deploy nodes across multiple zones based on multi-level heterogeneity, thereby enhancing both coverage and energy efficiency. The complexity of the network is increased when the CHs are increased. Therefore, three-level heterogeneity is introduced in [51]. Here, normal, advanced and super nodes are distributed in five separate zones. Although this does not bring a proper solution to the problem. Thus, Extended Z-SEP [52] was developed to eliminate this drawback by limiting the total quantity of CHs. The parent threshold value is changed to a residual energy-aware version. The CH selection threshold is derived as:


$$\begin{array}{c} T(n)=\left\{ \begin{array}{c} @l\frac{p}{\text{1}-p(rmod\frac{\text{1}}{p})}\times \frac{E_{res}}{E_{o}}\times K_{opt},\;ifn\in G\\ \text{0},\;otherwise \end{array}\right. \end{array}$$ (7)

Here, $K_{opt}$ is the optimal cluster number. If CHs deplete energy too quickly, instability may still occur, despite residual energy-based selection. Later on another modified hybrid protocol named Improved Zonal SEP (IZ-SEP) [53] is proposed where the number of neighbors of each node within the cluster is counted along with the residual energy for the CH selection. Here the node deployment is done in two separate zones: Zone 1 contains the normal nodes and Zone 2 contains the advanced nodes. The modified threshold used in this protocol is:


$$\begin{array}{c} T(n)\;=\left\{ \begin{array}{c} @l\frac{p}{\text{1}-p(rmod\frac{\text{1}}{p})}\times \frac{E_{res}(n)}{E_{avg}}\times \frac{{Nb}_{n}}{{Nb}_{avg}},\;ifn\in G\\ \text{0},\;otherwise \end{array}\right. \end{array}$$ (8)

Where ${Nb}_{n}$ represents the number of neighbors for node n and ${Nb}_{avg}$ is the average of all the neighboring nodes in the network. This improves the stability period of the network by choosing CH from the places where more nodes are deployed. But this also adds implementation complexity to the network. For clarity, Table 1 summarizes the major characteristics and limitations of existing routing protocols. The comparison demonstrates that none of the existing methods simultaneously address intelligent node deployment, network coverage optimization, and multi-level heterogeneity, which are the key contributions of the proposed EZS-SEP protocol.

**Table 1:** Comparison of existing routing protocols.

Ref.	Heterogeneity Level	Key Features	Limitations
[6]	Multi	Distance-aware residual energy routing with multi-hop communication	Does not address network coverage or intelligent node deployment
[9]	1	Randomized rotation of CH role using probability threshold	Does not support heterogeneous networks; ignores residual energy in CH election
[13]	2	Hybrid communication, residual energy- and distance-based CH selection, multi-hop routing	Does not optimize node deployment and is limited to two-level heterogeneity
[15]	2	Threshold-sensitive reactive routing with energy-aware CH election	Suitable mainly for event-driven applications
[16]	Multi	CH election based on residual and average network energy	Does not consider node location or zonal deployment
[17]	2	Weighted CH election based on initial energy	Does not consider residual energy, distance, or network coverage
[23]	2	Zone-based deployment with hybrid communication	Fixed zonal deployment and random CH selection; no energy-aware CH election
[33]	2	Distance-aware CH election based on different parameters, with sleep/awake mechanism	Does not consider multi-hop communication or dynamic topology changes
[37]	2	Energy-aware CH selection choosing highest-energy node among candidates with balanced cluster size	Does not consider residual energy, distance from BS, or multi-level heterogeneity
[38]	3	Distance- and energy-aware CH selection with optimal CH count	Does not optimize node deployment or network coverage
[39]	2	Uniform node distribution and improved CH selection criteria	Does not consider residual energy or coverage optimization
[49]	3	Dynamic CH election based on residual energy, average network energy, and optimal cluster count	Does not consider distance from BS; no multi-hop communication
[50]	3	Distance- and residual-energy-aware CH election with dual-hop communication for distant CHs	Performance degrades when BS is at the corner; no mobility support
[51]	3	Five-zone deployment with hybrid direct and multi-hop communication	Relies on static zones and lacks adaptive CH selection based on residual energy
[52]	2	Hierarchical clustering with residual-energy-based CH selection and hybrid communication	Does not consider network coverage or higher-level heterogeneity
[53]	2	CH selection using residual energy and neighbour density	Limited to two-level heterogeneity and does not optimize node placement

Reinforcement learning and machine learning techniques have recently attracted a lot of attention for improving WSNs. A reinforcement learning-based opportunistic routing protocol (RLOR) [54] was proposed for underwater acoustic sensor networks. It uses Q-learning to dynamically select relay nodes and avoid routing voids. This leads to better packet delivery ratio, reliability, energy efficiency, and shorter end-to-end delays. Soltani et al. [55] created a multi-agent reinforcement learning framework. In this setup, sensor nodes act as independent agents that learn energy-efficient routing strategies based on factors like residual energy, hop count, sink distance, and link quality, thereby improving network lifetime, load balancing, and routing flexibility in large-scale WSNs. Another study integrated reinforcement learning with a Hybrid Bat-Crow Search Algorithm (BCSA) [56] to jointly optimize routing and mobile sink relocation. The proposed mechanism dynamically determines forwarding paths and sink positions according to network conditions, leading to improvement in network performance. Aleem and Thumma [57] proposed a hybrid energy-efficient clustering framework that integrates K-Means clustering, the 0/1 knapsack algorithm, and reinforcement learning, where K-Means first partitions sensor nodes into clusters, the knapsack algorithm selects optimal CH candidates based on energy-related constraints, and reinforcement learning continuously refines the CH selection policy to maximize network lifetime and energy efficiency.

Total coverage is also an important issue while deploying the sensors since there will be blind areas in the network in case of gaps in the sensor locations. All these uncovered regions greatly undermine the quality of the data that is being gathered which may result in either inaccurate or inadequate analysis of the environment. Also, the decrease in the quality of data because of the partial coverage is even more troubling when applied to a delicate situation where its inadequacy may lead to some severe mishaps. Therefore, some methods have been studied like Distributed Self-Spreading Algorithm (DSSA) [58]. It employs a synergistic blend of peer-to-peer deployment and cluster structuring. But several DSSA assumptions are not relevant in the real-world scenario, including sensitivity to the minimal percentage of initial coverage, initial uniformity and non-single-point deployment. Therefore, Self-Organizing Node Deployment Algorithm (SODA) [59] was proposed to address deployment inefficiencies. Inspired by molecular equilibrium, SODA rapidly structures nodes even when they are randomly placed by adjusting movement with respect to local density. Kohonen Self-Organizing Maps (SOMs) [20] can be utilized to optimize sensor node deployment by preserving the topological relationships among nodes, thereby improving network coverage, connectivity, and load distribution while reducing coverage holes. The distributed SOM approach proposed in [60] extends the classical Kohonen SOM by enabling decentralized node localization without relying on a central controller, improving scalability and reducing computational complexity for wireless sensor networks. This demonstrates the potential of SOM-based techniques to enhance both network coverage and localization efficiency in WSN applications. Another approach introduced in [22] where Hebbian machine learning was used for node deployment. Here the sensors are deployed randomly and transmit data to a central sink that learns a Hebbian model per sensor using second last data. The resulting weight vectors are remembered and are utilized to group nodes regionally. Subsequently, Smart Self-organizing Node Deployment (SSND) [61] was proposed. It maximizes the coverage and uses minimal amount of energy by covering one sensor per neighborhood at a time. Hoang et al. [21] proposed a SOM-based UAV deployment framework in that dynamically optimizes aerial BS placement to maximize network area coverage and population connectivity in disaster-affected regions. A comparative summary of different deployment methods, including their mechanisms, key features, and computational complexities, is presented in Table 2, where n is the number of sensor nodes, e is the number of SOM training epochs, and k is the number of deployment iterations.

**Table 2:** Comparison of SOM with other deployment methods.

Ref.	Deployment Method	Mechanism	Key Features	Computational Complexity
[22]	Hebbian-based SOM	Uses a Kohonen SOM trained with Hebbian competitive learning to determine sensor locations	Topology-preserving, intelligent deployment	O(ne)
[58]	Distributed Self-Spreading Algorithm (DSSA)	Nodes iteratively spread using local neighborhood information until equilibrium is reached	Distributed deployment, balanced coverage	$O(kn^{\text{2}})$
[59]	Self-Organized Deployment Algorithm (SODA)	Nodes self-organize through local interactions and coverage optimization	Self-organizing, distributed operation	$O(kn^{\text{2}})$
[21]	Self-Organizing Map (SOM)	Employs a SOM to optimize node placement for maximum sensing coverage	Improved coverage, reduced coverage holes	O(ne)
[60]	Distributed Self-Organizing Map (SOM)	Extends the classical SOM into a distributed learning framework for node localization	Distributed learning, scalable localization	$O(n^{\text{2}})$

Compared with existing deployment techniques, the Self-Organizing Map (SOM) provides a topology-preserving deployment that distributes sensor nodes more uniformly, thereby reducing coverage holes and improving network connectivity. Unlike DSSA and SODA, which require iterative node relocation and repeated neighborhood interactions, the SOM performs a single training phase which does not incur additional communication overhead and complexity during network operation. Moreover, the learned node positions can be directly mapped into heterogeneous zones, making SOM particularly suitable for the proposed protocol by achieving balanced energy consumption and extending the network lifetime.

In this article, we propose EZS-SEP, a proactive three-level heterogeneous routing protocol that considers both node energy and node position during CH selection. The main objective of the proposed protocol is to extend the stability period, improve throughput and increase the overall network lifetime. EZS-SEP selects CHs by incorporating residual energy, average network energy and distance from the base station, which helps prevent low-energy nodes from being selected as CHs and supports more balanced energy consumption across the network. In addition, the protocol establishes a three-level heterogeneous network and uses a self-organizing map for proper node distribution, ensuring improved network coverage and more reliable data transmission. Furthermore, EZS-SEP employs a hybrid communication mechanism to support efficient energy usage among nodes, thereby enhancing the overall performance and reliability of the wireless sensor network.

## 3. System models

The proposed routing protocol represents an enhanced version of the Z-SEP protocol, incorporating improved CH selection and energy-aware communication mechanisms to achieve better stability and prolonged network lifetime. To realize the proposed scheme, a heterogeneous network model and a radio energy dissipation model have been adopted. A brief description of each model is presented in the following section.

### 3.1. Energy dissipation model

**Fig 2:**
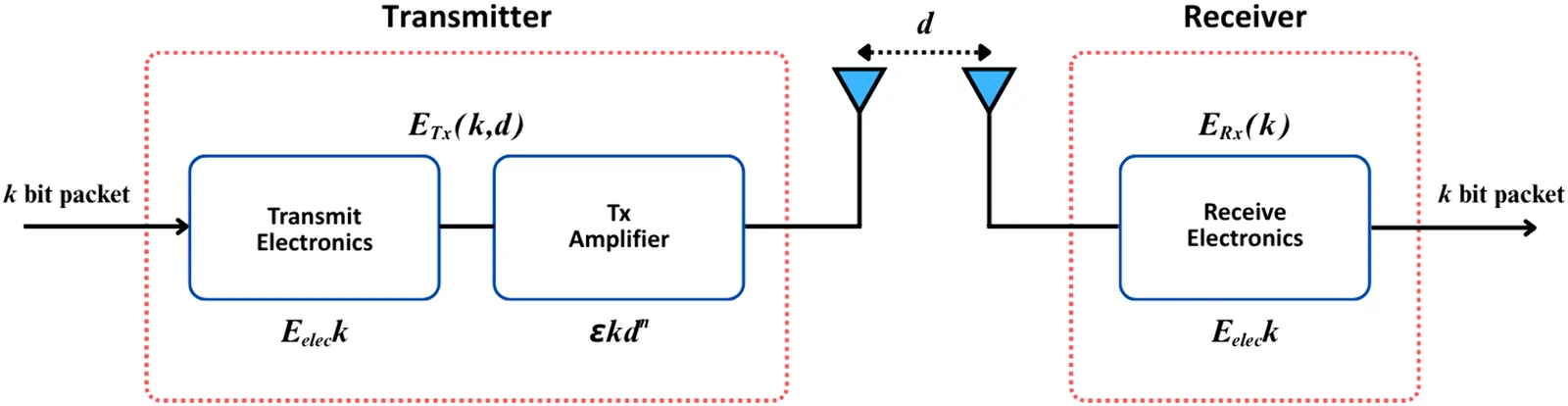
Energy dissipation model.


The energy dissipation model illustrated in Fig 2 is adopted in the proposed protocol to characterize data transmission and reception processes. In WSNs, data transmission consumes significantly more energy than data reception [62]. In this model, both the transmitter and receiver contribute to the overall energy dissipation in a wireless communication system. Energy is used by the transmitter to run the power amplifier which amplifies the signal for transmission and also the radio circuits. The energy dissipation increases with the separation distance, d, as the power amplifier compensates for the signal loss during propagation. The receiver, on the other hand, consumes energy to operate its radio electronics, which are responsible for receiving and processing the transmitted signal. The energy consumption at the receiver is typically constant but is still crucial to the overall energy efficiency. The model also takes into account the separation distance, d, between the transmitter and receiver, where energy dissipation increases as the distance grows due to path loss. To mitigate this, the power amplifier at the transmitter boosts the signal strength to compensate for the loss, ensuring that the receiver can accurately receive the signal even over long distances. This amplification process helps maintain reliable communication by counteracting the natural attenuation of the signal as it travels through space, thus balancing the energy required for both transmission and reception. When transferring a k-bit data packet to a sensor node d distance away, the power required can be calculated as [39]:

$$\begin{array}{c} E_{Tx}(k,d)≜\left\{ \begin{array}{c} @lk\left(E_{elec}+\epsilon_{fs}d^{\text{2}}\right),\;ifd\leq d_{o}\\ k\left(E_{elec}+\epsilon_{mp}d^{\text{4}}\right),\;ifd>d_{o} \end{array}\right. \end{array}$$ (9)

Here, d is the Euclidian distance between the sender and the receiver node. It is to be noted that $E_{elec}$ is the energy cost per bit for the transmitter or receiver circuit, ε_*fs*_ and ε_*mp*_ are the transmission parameters for the free space and the multipath fading models respectively. The reference distance is $d_{o}$ and it is calculated as:


$$\begin{array}{c} d_{o}≜\sqrt{\frac{\epsilon_{fs}}{\epsilon_{mp}}} \end{array}$$ (10)

In addition, it is possible to calculate the energy usage for receiving k-bit data using,


$$\begin{array}{c} E_{Rx}≜kE_{elec} \end{array}$$ (11)

### 3.2. Heterogeneous Network Model

**Fig 3:**
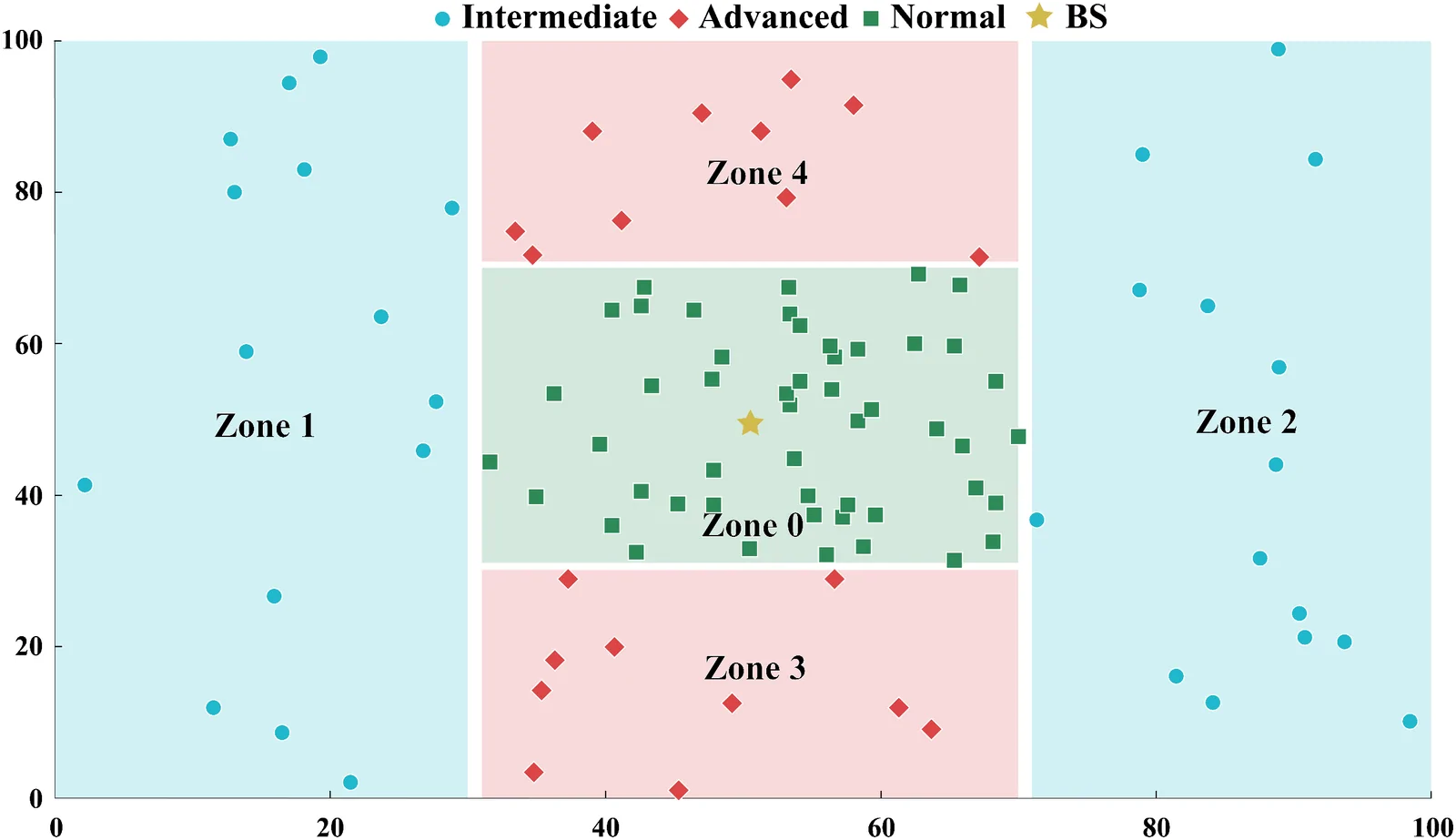
The proposed network architecture.

An essential component of the network’s optimal functioning is the energy heterogeneity of its nodes. In WSN, different levels of node heterogeneity is considered. In this article, three different node types: normal, intermediate and advanced have been deployed in a heterogeneous network of n nodes. The proposed network architecture is illustrated in Fig 3.

We have assumed that m and $m_{o}$ are the percentage of nodes that are advanced nodes and intermediate nodes. Compared to normal nodes, the advanced and intermediate nodes possess α and β times more energy, respectively. The relation between α and β is β=α/2. Consequently, there are n×m advanced nodes, ${n\times m}_{o}$ intermediate nodes and $n\times (\text{1}-m-m_{o})$ normal nodes. With the normal, intermediate and advanced nodes’ initial energies represented as $E_{nrm}$, $E_{int}$ and $E_{adv}$ respectively, the energy relationships may be obtained by,


$$\begin{array}{c} E_{nrm}=E_{o} \end{array}$$ (12)

$$\begin{array}{c} E_{int}=E_{o}(\text{1}+\beta ) \end{array}$$ (13)

$$\begin{array}{c} E_{adv}=E_{o}(\text{1}+\alpha ) \end{array}$$ (14)

The total energy of the network can be calculated by,


$$\begin{array}{c} E_{Total}=n\left(\text{1}-m-m_{o}\right)\times E_{nrm}+nm_{o}\times E_{int}+nm\times E_{adv} \end{array}$$

$$\begin{array}{c} =n\left(\text{1}-m-m_{o}\right)\times E_{o}+nm_{o}\times E_{o}(\text{1}+\beta )+nm\times E_{o}(\text{1}+\alpha ) \end{array}$$

$$\begin{array}{c} =n\times E_{o}(\text{1}+\text{m}\alpha +m_{o}\beta ) \end{array}$$ (15)

## 4. The proposed protocol

In this work, we have proposed a new protocol called EZS-SEP to enhance energy efficiency and stability in heterogeneous WSN environments. In EZS-SEP, heterogeneous nodes participate in hybrid communication. Advanced and intermediate nodes function as CHs to collect data from the adjacent nodes and send it to BS. Meanwhile normal nodes do not become CHs and communicate directly with BS. Depending on their energy, these nodes are placed in particular zones. Intermediate and advanced nodes are positioned farther from the BS, while normal nodes are positioned closer to the BS. This strategy promotes more balanced energy distribution throughout the network field. The CH selection process of EZS-SEP with advanced and intermediate nodes take into account five major factors: (1) residual node energy, (2) initial node energy, (3) distance to the BS, (4) average distance from the BS of alive nodes and finally (5) the average residual energy of all alive nodes. This multi-parameter approach enables more informed CH selection, promoting uniform energy dissipation and prolonged network lifetime.

### 4.1. Node Deployment Strategy

**Fig 4:**
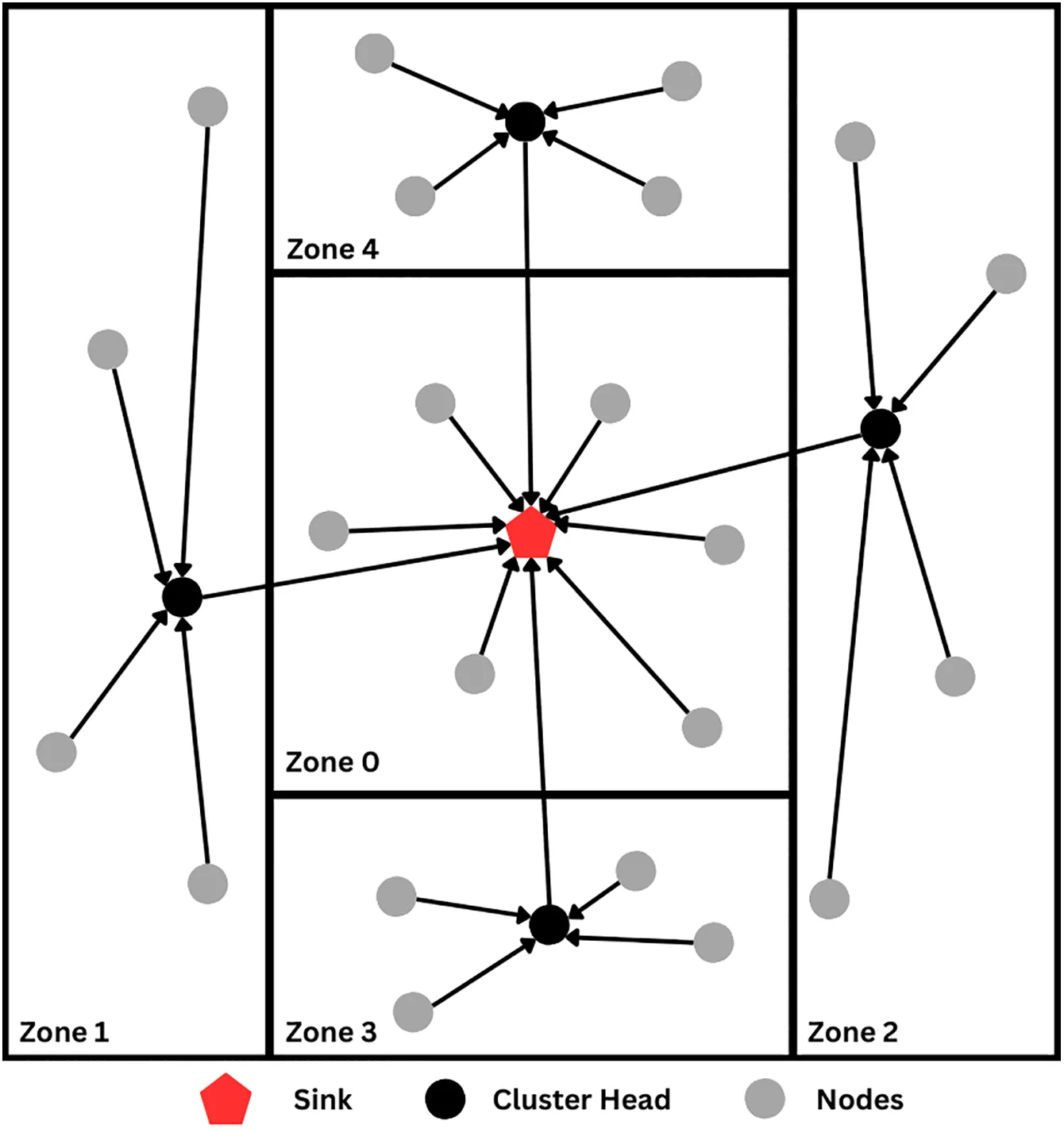
Data communication architecture between sensor nodes and BS.

In our proposed protocol, we have considered that all the nodes are static and the BS is located in the middle of the field. The nodes are set up using a unique technique. We have used Self-organizing maps for the deployment of the nodes. There are five zones in the entire network region (Zone 0–4) as displayed in Fig 4. Normal nodes are equipped with lower initial energy and that is why they are placed in the Zone 0, closer to the BS. While Zone 1 and 2 have intermediate nodes, whereas Zones 3 and 4 have advanced nodes. As the distance from the BS rises, so does the amount of energy consumption, this is the reason behind this kind of node deployment. The normal nodes are not involved in the clustering process and communicate with the BS directly. Whereas, intermediate and advanced nodes form clusters for CH selection and after the selection of CH, data is gathered by CH from the subordinate nodes, aggregated and then sent to the sink.

SOM was selected for node deployment because of its ability to preserve the topological structure of the input space while producing an organized and well-distributed node layout. Unlike purely random deployment, SOM minimizes node clustering and coverage holes by distributing sensor nodes more uniformly throughout the sensing field. This results in improved network coverage, enhanced connectivity, balanced node density, and shorter communication distances between neighboring nodes and CHs. Consequently, the proposed deployment strategy contributes to more balanced energy consumption, prolonged network stability, and an extended network lifetime.

This node deployment uses Self-Organizing Map (SOM), where hexagonal grids are used to map data points based on similarity. SOM creates a 10×10 grid and trains it using random input data. After training, node positions are extracted and randomizes the zone assignments by shuffling the input data points. The goal is to organize nodes into zones. Each hexagonal cell in the grid corresponds to a specific data point or group of similar data points and the numbers inside the hexagons (e.g., 1, 2, 3) represent the frequency or “hits” of how many times a particular data point has been mapped to that cell. The color intensity of the hexagons corresponds to the number of hits, with darker shades indicating higher frequencies and lighter colors indicating fewer hits.

The SOM introduces only a one-time initialization memory overhead proportional to the number of neurons and input vectors used during training. Specifically, memory is temporarily allocated for the neuron weight vectors, neighborhood topology, learning-rate parameters, and input coordinate matrix during the offline training process. Since node deployment is completed before network operation begins, these temporary data structures are released after convergence, and only the final node coordinates are preserved. As a result, the proposed protocol does not require storing the SOM model, neuron weights, or any additional inference-related data during runtime. Consequently, the operational memory complexity remains essentially the same as conventional SEP-based protocols, while the SOM overhead is confined to the preprocessing stage.

### 4.2. Hybrid communication

A hybrid communication has been developed for the data transmission to the BS where the normal nodes interact directly with the BS (direct communication), while advanced and intermediate nodes use CH to communicate with the BS.

#### 4.2.1. Direct communication.

Normal nodes are positioned in Zone 0, which is in close proximity to the BS. Once the data is collected, the normal nodes process the information and directly transmit it to the BS for further analysis or processing. Due to their proximity to the BS, these nodes have shorter transmission distances, resulting in reduced energy consumption during data transmission. Their role is crucial for real-time data collection and communication with the central system, ensuring efficient data flow.

#### 4.2.2. Communication via cluster head.

Zones 1 and 2 house intermediate nodes, whereas Zones 3 and 4 house advanced nodes. These nodes use CHs to transmit their data to the BS. For choosing CHs, there are numerous algorithms available. With the SEP [17] algorithm being one of the most well-known. SEP is a proactive clustering protocol that forms several clusters, each with one CH, which changes every round. The CHs use data aggregation techniques and transmit the collected data directly to the BS, helping to reduce the energy consumption of sensor nodes. However, the main drawback of SEP is its random CH selection process, which does not consider any important factors like nodes residual energy or distance from BS. Due to these limitations, particularly the lack of key considerations during CH selection, we introduced a new protocol that improves CH election. In our protocol, we counted the energy and distance factors, giving nodes nearer the BS and with greater energy an increased likelihood of getting chosen as CH in every round.

**4.2.2.1 Nodes’ Weighted Probabilities:** Let p be the node selection probability, which is used to select intermediate and advanced nodes as a CH in the $r^{th}$ round. $P_{int}$ and $P_{adv}$are normalized probability values for intermediate and advanced nodes. Initial node energy, average node energy, average node distance from BS and average node distance from BS are used to calculate the weight. This weight allows a larger probability to be high for more energized nodes. The weighted intermediate and advanced nodes’ probabilities can be written as,


$$\begin{array}{c} P_{int}=\frac{p(\text{1}+\beta )}{\text{1}+\alpha m+\beta m_{o}}\times \frac{E_{avg}(r)}{E_{ii}}\times \frac{D_{avg}(r)}{D_{i}} \end{array}$$ (16)

$$\begin{array}{c} P_{adv}=\frac{p(\text{1}+\alpha )}{\text{1}+\alpha m+\beta m_{o}}\times \frac{E_{avg}(r)}{E_{ai}}\times \frac{D_{avg}(r)}{D_{i}} \end{array}$$ (17)

Where $E_{ii}$ and $E_{ai}$ is the initial energy of the intermediate and advanced nodes, $D_{i}$ is the nodes distance from BS, $E_{avg}(r)$ is the total average energy of all alive nodes and $D_{avg}(r)$ is the average distance of all alive nodes from the BS. $E_{avg}(r)$ and $D_{avg}(r)$ can be expressed by,


$$\begin{array}{c} E_{avg}(r)=\frac{\text{1}}{N}\sum_{i=\text{1}}^{N}E_{N_{i}}(\text{r}),\text{ if }E_{N_{i}}(\text{r})>\text{0} \end{array}$$ (18)

$$\begin{array}{c} D_{i}=\sqrt{{\left(X_{i}-X_{bs}\right)}^{\text{2}}+({Y_{i}-Y_{bs})}^{\text{2}}} \end{array}$$ (19)

$$\begin{array}{c} D_{avg}(r)=\frac{\text{1}}{N}\sum_{i=\text{1}}^{N}D_{i} \end{array}$$ (20)

Here, $\left(X_{i},Y_{i}\right)$ and $\left(X_{bs},Y_{bs}\right)$ are the coordinates of the $i^{th}$ node and the sink respectively. N is the number of alive nodes in that round which is equivalent to n prior to the death of the first node, $E_{N_{i}}(r)$ is the nodes remaining energy in $r^{th}$ round and $D_{i}$ is the distance between the node and the sink.

**4.2.2.2 Threshold Criteria for CH Selection:** Throughout the CH selection process, every node produces a random number between 0 and 1. If this value falls below the threshold, which is denoted by $T_{int}$ for intermediate and $T_{adv}$ for advanced nodes, then it will be selected as CH. The newly enhanced threshold formula is then obtained as:


$$\begin{array}{c} T_{int}=\left\{ \begin{array}{c} @l\frac{P_{int}}{\text{1}-P_{int}\left(rmod\frac{\text{1}}{P_{int}}\right)}\times \frac{E_{res}}{E_{avg}(r)}\times \frac{D_{avg}(r)}{D_{i}},\;ifN_{i}\in G^{\prime }\\ \text{0},\;otherwise \end{array}\right. \end{array}$$ (21)

$$\begin{array}{c} {\;T}_{adv}=\left\{ \begin{array}{c} @l\frac{P_{adv}}{\text{1}-P_{adv}(rmod\frac{\text{1}}{P_{adv}})}\times \frac{E_{avg}(r)}{E_{res}}\times \frac{D_{avg}(r)}{D_{i}},\;ifN_{i}\in G^{\prime \prime }\\ \text{0},\;otherwise \end{array}\right. \end{array}$$ (22)

Here, $P_{int}$, $P_{adv}$, $E_{avg}(r)$, $D_{avg}(r)$, $D_{i}$ and r is same as before. $G^{\prime }$is a collection of intermediate nodes that has not been selected as CH in the previous $\frac{\text{1}}{P_{int}}$ rounds and $G^{\prime \prime }$ is the set of advanced nodes that has not been CH in the last $\frac{\text{1}}{P_{adv}}$ rounds. $E_{res}$is the residual energy of that $i^{th}$ node.

### 4.3. Implementation Phases of EZS-SEP

The lifetime of WSN is highly reliant on energy efficiency and stability period. EZS-SEP meets these objectives by selecting the CH in an efficient manner. This algorithm works in a sequence of defined stages for optimum data relay and clustering. EZS-SEP operates in conjunction with two main phases during each round: the set-up phase and steady-state phase, which is same as the traditional LEACH [9] and SEP [17] protocol.

**Fig 5:**
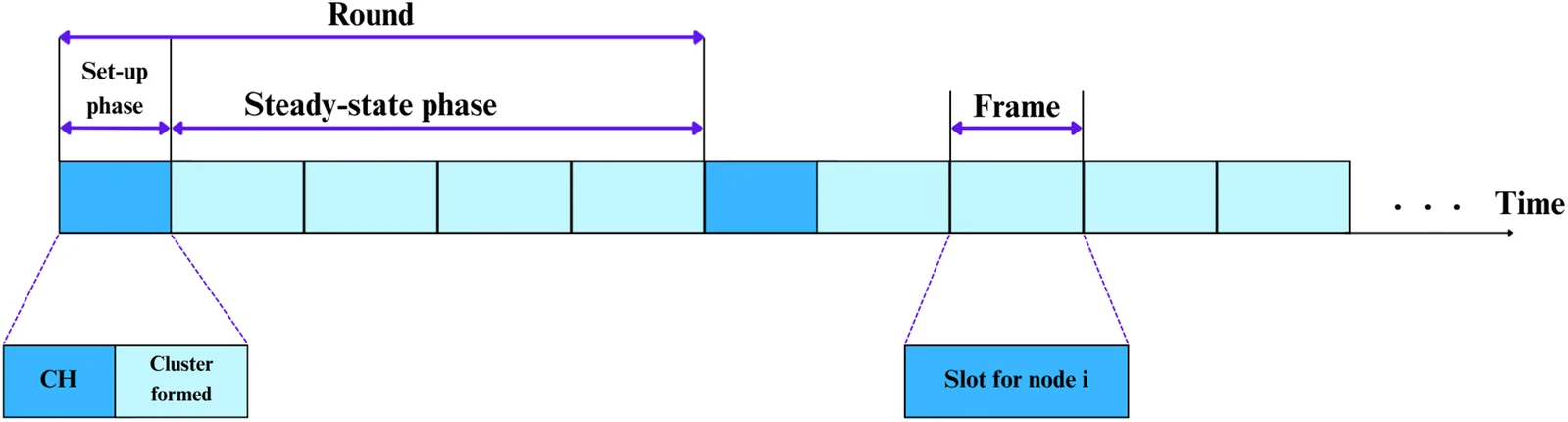
Implementation phases of EZS-SEP protocol.

All of these phases are critical to the overall performance of the network, including the initial CH selection and the continuous data aggregation and transmission to the BS. In this section, we describe the implementation phases of EZS-SEP: how clusters are formed and organized, how nodes within a cluster interact with one another, and how data is communicated efficiently to ensure proper processing while conserving energy and maintaining network stability. Fig 5 illustrates the general methodology of these stages.

**Set-Up Phase**: A CH is chosen in the initial CH selection depending on threshold function equation. After that, the CH sends out an advertisement to nodes through CSMA MAC which invites them for cluster formation. When the advertisement is received, each member node looks for the CH with stronger signal to join. After the member nodes have joined the cluster, they inform the CH of their arrival. The CH assigns a TDMA schedule to control data transmission and avoid collisions among nodes.

**Steady State Phase**: During this stage, data is sent to CH by the member nodes according with time slots assigned in last phase. The CH communicates with each node for data aggregation, aggregates the data from each node to avoid redundancy of information and compresses it for efficient utilization of available energy. Finally, the CH forwards the aggregate data to the BS.

**Fig 6:**
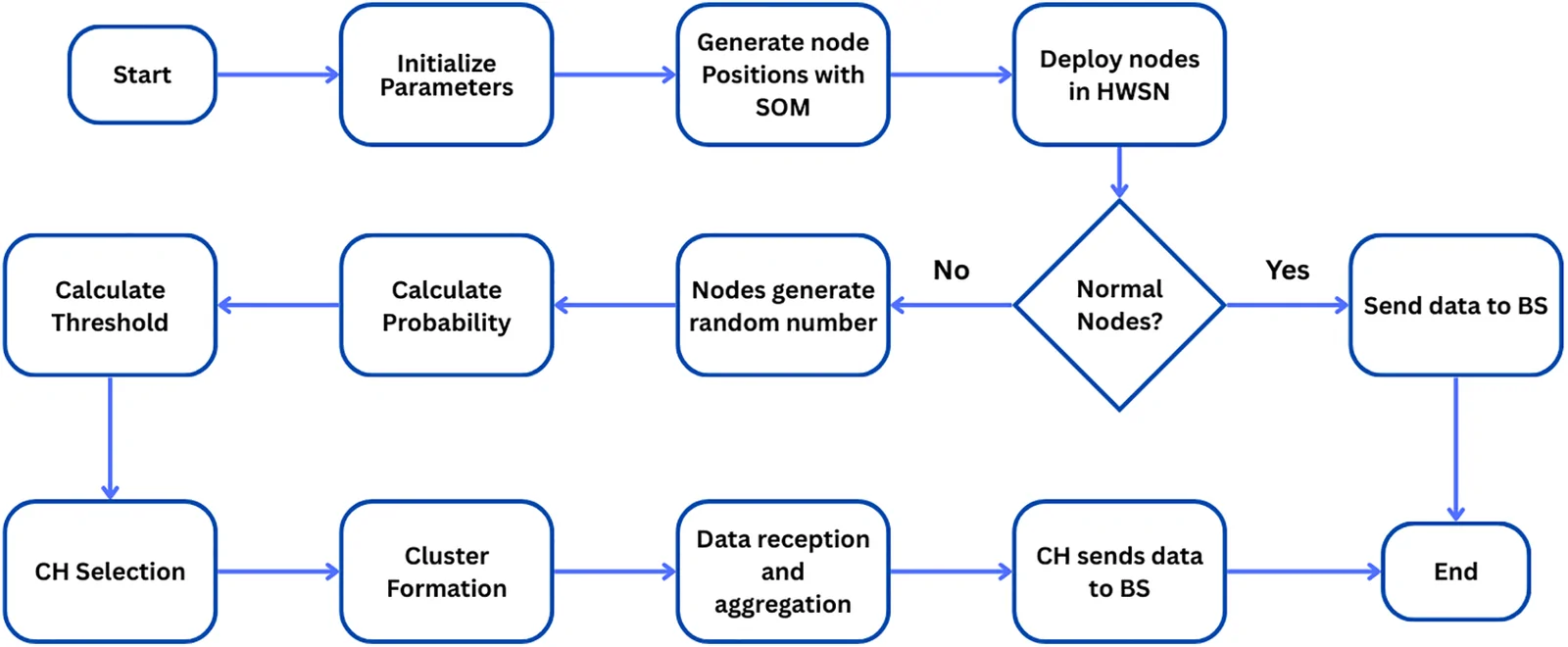
Flowchart of EZS-SEP.

This is done repeatedly and multiple times in rounds which is energy efficient multipartite communication, offering the benefits of traffic scheduling (TDMA) to avoid packet collision and aggregated data for reducing the consumption power as well as maximizing the data transmission efficiency. From Fig 6, the complete process of the proposed method can be observed. The node positions are generated using SOM, after which they are deployed into separate zones according to their respective energy levels. A specific condition is considered whereby normal nodes do not participate in the CH selection process and instead transmit data packets directly to the BS. This implies that normal nodes located closer to the sink communicate directly with the BS without the assistance of a CH. On the other hand, intermediate and advanced nodes undergo a CH selection procedure. Each node generates a random number between 0 and 1, after which the respective probabilities $\left(P_{adv},P_{int}\right)$ are calculated. Subsequently, the threshold value for each node is determined. The generated random number is then compared with the calculated threshold; if the threshold exceeds the random number, the node is elected as a CH. Once selected, the CH broadcasts an advertisement message to neighboring nodes to join its cluster. After cluster formation, the CH collects data from its member nodes, performs data aggregation, and transmits the compiled information to the BS. Through this hybrid communication mechanism, network lifetime is prolonged and energy consumption is efficiently managed. Algorithm 1 includes all the steps related to the proposed protocol.

The proposed multi-factor CH selection introduces negligible memory overhead, as each sensor node stores only a few additional scalar parameters, including residual energy, initial energy, distance to the BS, average network energy, and the CH election threshold. No routing tables, optimization populations, or machine learning models are maintained during runtime, resulting in a constant per-node memory complexity of O(1) and an overall network memory complexity of O(N), comparable to conventional SEP-based protocols.

EZS-SEP introduces negligible communication overhead compared with other SEP-based protocols because the SOM-based node deployment is executed only once during the initialization phase and does not require additional control packet exchanges during network operation. Thereafter, EZS-SEP follows the same clustering, CH advertisement, cluster formation, and data transmission procedures as conventional SEP-based protocols. Therefore, the communication overhead per communication round remains comparable to that of the baseline protocols.

**Algorithm 1.** EZS-SEP Based Routing

CH_set: Set of CH

CH_count: CH counter

Packets_to_BS: Packets sent to base station

$N_{rand}$: Random number

$N_{nrm}$: Set of normal nodes

$N_{int}$: Set of intermediate nodes

$N_{adv}$: Set of advanced nodes

SOM: Self-organizing map

1: **begin**

2: Take random input to build SOM

3: Train SOM with random input

4: Extract positions and build sensor network

5: **for** r = 1 to $r_{max}$ **do**

6:  CH_set = 0

7:  CH_count = 0

8:  **Calculate** $P_{int}$, $P_{adv}$, $E_{avg}(r)$, $D_{i}$, $D_{avg}(r)$ by using Eq. (16)-(20);

9:   **for** i = 1 to n **do**

10:   **if**($\left\{N_{i}\right\}\in N_{nrm}\&E_{N_{i}}(r)>\text{0}$) **then**

11:    Normal node $N_{i}$ sends data directly to the BS;

12:    Packets_to_BS = Packets_to_BS + 1;

13:    **Calculate** $E_{Tx}(k,d)$ by using Eq. (9) and **Update** $E_{N_{i}}(r)$;

14:   **end if**

15:    **Calculate** $T_{int}$, $T_{adv}$ by using Eq. (21), (22);

16:   **if**(${\{N}_{i}\}\in N_{int}\&N_{rand}<T_{int}\&E_{N_{i}}(r)>\text{0}\&G^{\prime }>\text{0}$) **then**

17:    Intermediate node $N_{i}$ is selected as CH;

18:    CH_set = CH_set$\cup \{N_{i}\}$;

19:    CH_count = CH_count + 1;

20:    Packets_to_BS = Packets_to_BS + 1;

21:    **Calculate** $E_{Tx}(k,d),E_{Rx}$ by using Eq. (9), (11) and **Update** $E_{N_{i}}(r)$;

22:   **end if**

23:   **if**(${\{N}_{i}\}\in N_{adv}\&N_{rand}<T_{adv}\&E_{N_{i}}(r)>\text{0}\&G^{\prime \prime }>\text{0}$) **then**

24:    Advanced node $N_{i}$ is selected as CH;

25:    CH_set = CH_set$\cup \{N_{i}\}$;

26:    CH_count = CH_count + 1;

27:    Packets_to_BS = Packets_to_BS + 1;

28:    **Calculate** $E_{Tx}(k,d),E_{Rx}$ by using Eq. (9), (11) and **Update** $E_{N_{i}}(r)$;

29:    **end if**

30:   **end for**

31: **end for**

32: **end**

## 5. Simulation and result analysis

In this section, we critically evaluate key WSN performance parameters by comparing the proposed EZS-SEP protocol with existing schemes such as ESEP, P-SEP, Z-SEP, and LEACH under identical simulation conditions. The comparison is conducted in terms of node death rate, throughput, total energy consumption throughout the simulation, and average residual energy per node. This section presents comprehensive simulation results and tabulated analyses to highlight the performance differences among the protocols. By examining these metrics, a detailed assessment is provided regarding the strengths and limitations of each protocol, particularly in terms of energy efficiency and network lifetime enhancement in WSNs.

### 5.1. Simulation Setup

A simulation framework developed with MATLAB, version 2023a, is used to implement the network and evaluate its performance. The size of the network is 100m×100m, in which there are 100 heterogeneous sensor nodes. The network has a stationary BS at the center of it, which is equipped with an infinite amount of energy. After simulation in MATLAB, we have used Origin Pro 2024 for plotting the results and graphical comparison with existing protocols. Table 3 illustrates the network parameters employed in the simulation model.

**Table 3:** Parameters settings.

Parameters	Value
Network size,M	100m × 100m
Number of sensor nodes,n	100
Packet length,k	4000bits
Transmitter/Receiver electronics energy,$E_{elec}$	50nJ/bit
Initial energy of normal nodes,$E_{o}$	0.5J
Data aggregation energy,$E_{DA}$	5nJ/bit
Data transmission energy,$E_{TX}$	5bJ/bit
Energy dissipation for free space model,$\varepsilon_{fs}$	10pJ/bit/m^2^
Energy dissipation for multipath model,$\varepsilon_{mp}$	0.0013pJ/bit/m^4^
Optimal probability,p	0.1
Additional energy factor for advanced nodes,α	3
Additional energy factor for intermediate nodes,β	1.5
Fraction of advanced nodes,m	0.2
Fraction of intermediate nodes,$m_{o}$	0.3

### 5.2. Performance Metrics

Performance metrics are quantitative measures used to analyze and evaluate a protocol or network in terms of overall performance, effectiveness, and efficiency.

**Stability period:** The number of rounds from the commencement of network operation to the death of the first sensor node that is referred to as time period when a sensor node passes all its energy. This metric is crucial for assessing the energy efficiency and initial stability of the network.

**Network Lifetime:** The time the total round from beginning to death of last alive nodes, i.e., when network has died following that the node will participate no more. This connection availability is very important when evaluating the network’s total lifespan and energy efficiency or in other words how long a wireless network could be alive.

**Half-node death (HND):** The number of rounds required until 50% of the sensor nodes deplete their energy and become inactive. This metric indicates how effectively the protocol manages energy distribution before a significant portion of the network fails.

**Number of alive nodes per round:** The number of alive nodes per round refers to the nodes that have not depleted their energy and are still participating in data transmission.

**Number of packets to BS:** The total number of packets sent to the BS throughout the whole simulation.

**Area coverage:** Area coverage denotes the maximum size of physical space that a WSN can be used or sensed. It is an essential index to assess the network performance of WSNs, which reflects the ability that WSN can monitor and collect data in the monitoring area.

### 5.3. Simulation results and comparative analysis

In this protocol, nodes are deployed using a SOM-based approach to improve area coverage. Area coverage data were recorded for nodes with different sensing capabilities under both SOM-based deployment and without SOM deployment. The obtained results were then compared with ESEP, P-SEP, Z-SEP, and LEACH. The comparison metrics include the number of alive nodes per round, dead nodes per round, packets transmitted to the BS, and residual energy remaining per round under varied network parameters. Additionally, dynamic network conditions were considered by modifying network settings, such as changing the BS location, varying the initial energy level and changing the network size. The robustness of the proposed protocol was evaluated under these varying conditions.

#### 5.3.1. Area coverage.

**Fig 7:**
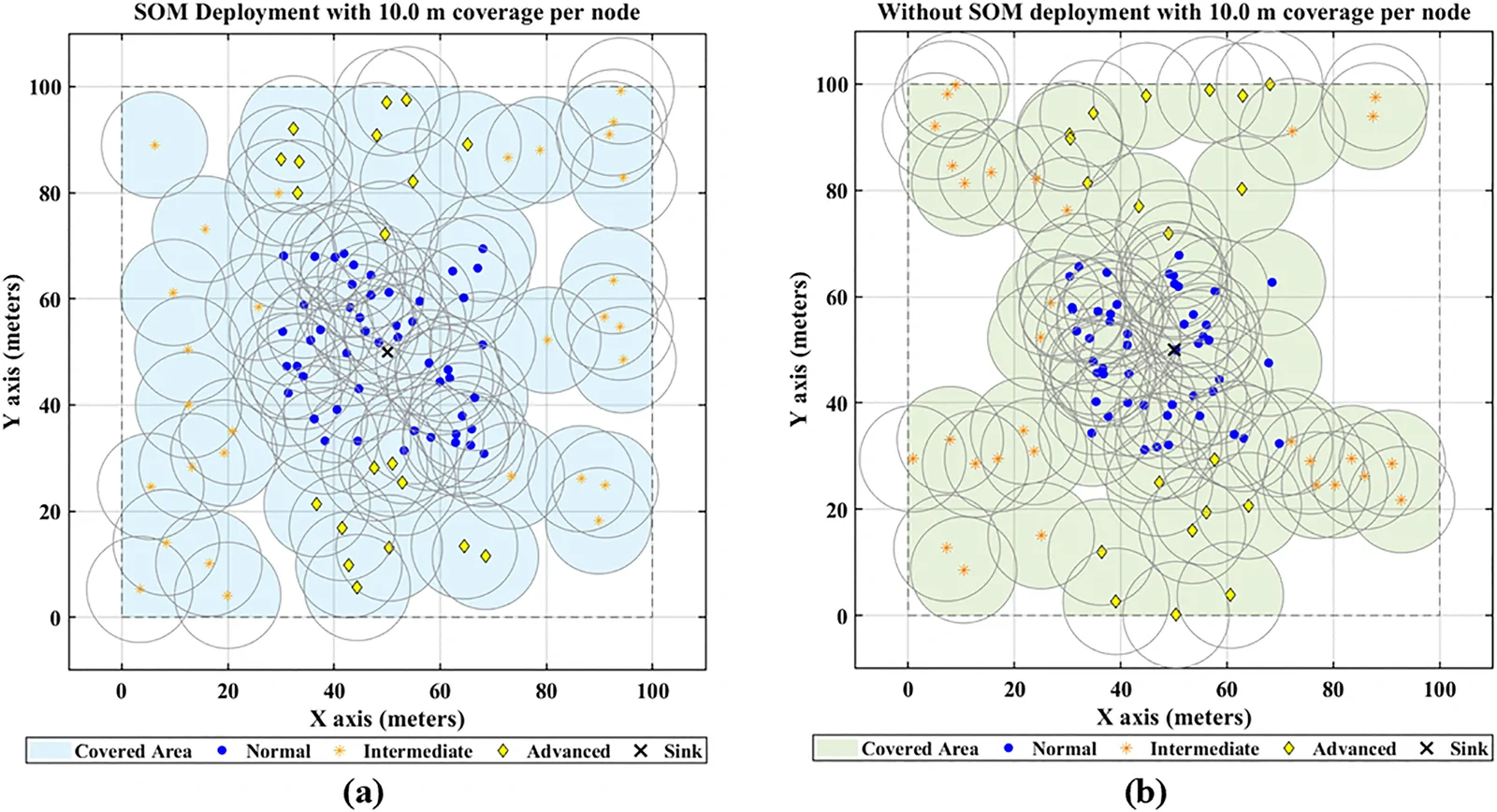
Node deployment (a) with SOM and (b) without SOM.

The nodes were deployed under two scenarios, with SOM-based deployment and without SOM, where the nodes had varying sensing ranges from 5 m to 10 m. The simulation’s output is displayed in Fig 7 and Table 4 reveals the overall improvement after this approach.

**Table 4:** Area coverage comparison with and without SOM.

		With SOM		Without SOM		Improvement
Radius (m)	Area (100×100 m^2^)	Covered Area (m^2^)	Percentage (%)	Covered Area (m^2^)	Percentage (%)	Percentage (%)
5		4770.9093	47.70%	4276.7539	42.77%	4.93%
6		5677.407	56.77%	5185.9977	51.86%	5.91%
7	10000	6975.2184	69.75%	6352.8875	63.53%	6.22%
8		7688.5433	76.88%	7123.9009	71.23%	5.65%
9		8246.9534	82.47%	7750.3234	77.50%	4.97%
10		8945.9786	89.46%	8259.2968	82.59%	6.87%

We have considered that each node senses a particular radius of area and then counted the total area covered by all the nodes. SOM achieves a more balanced deployment of sensor nodes, reducing coverage holes while improving overall sensing coverage. Fig 7 clearly shows that the deployment using SOM covers much more area than the deployment without SOM. This way we can find the overall area coverage, more the coverage by the nodes, more the data reliability. It is evident that for different node sensing range SOM deployment covers more area than traditional deployment. There is about 5% to 7% improvement in the overall network coverage.

#### 5.3.2. Number of Alive nodes.

**Fig 8:**
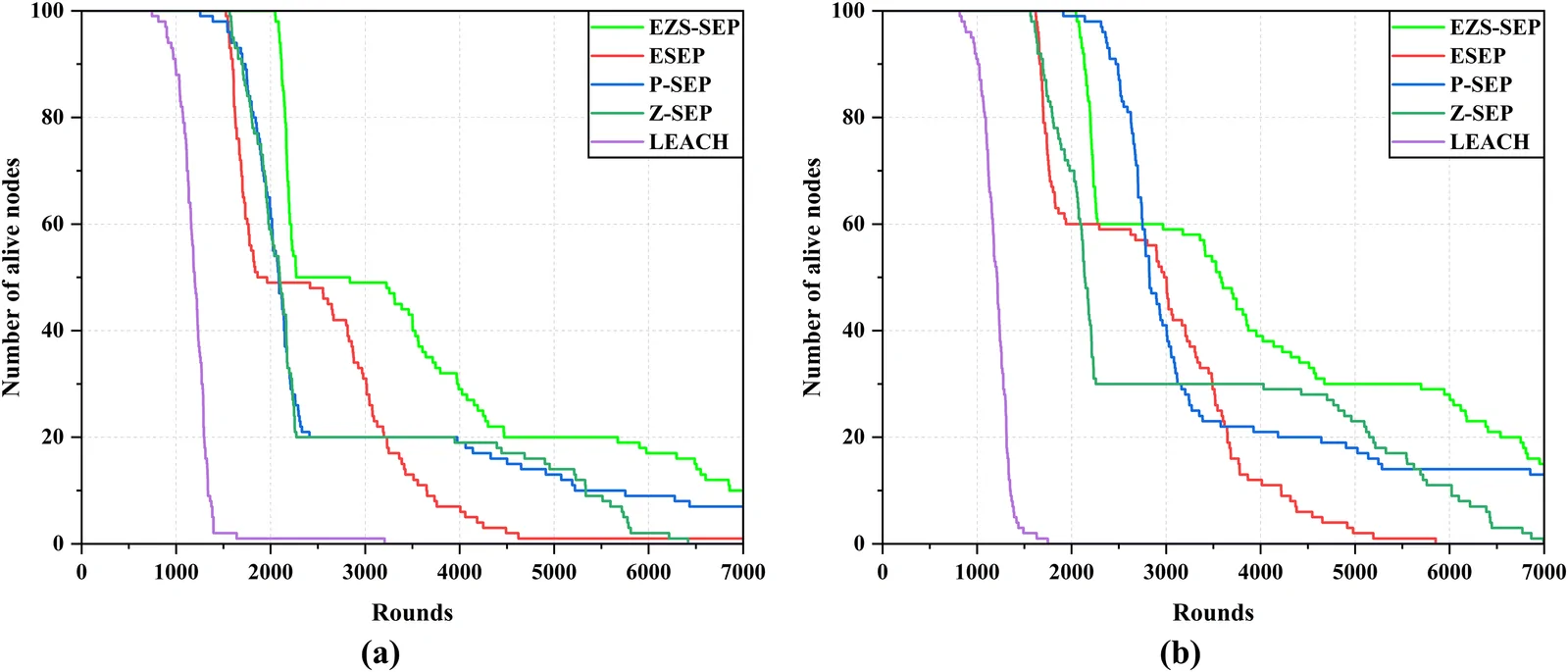
Alive nodes per round for (a) $m=0.2,m_{o}=0.3$ and (b) $m=0.2,m_{o}=0.3$.

Fig 8 compares our protocol with other existing methods under two distinct scenarios (a) $m=\text{0}.\text{2},m_{o}=\text{0}.\text{3}$ and (b) $m=\text{0}.\text{3},m_{o}=\text{0}.\text{3}$. It can be observed that the proposed protocol maintains a higher number of alive nodes per round compared to the other protocols. For case (a), the FND values are 2049, 1531, 1256, 1567, and 746 for EZS-SEP, ESEP, P-SEP, Z-SEP, and LEACH, respectively. This corresponds to approximately 34%, 63%, 31%, and 174% improvement in stability period compared to ESEP, P-SEP, Z-SEP, and LEACH, respectively. For case (b), the FND values are 2048, 1622, 1914, 1566, and 817 for EZS-SEP, ESEP, P-SEP, Z-SEP, and LEACH, respectively. In this scenario, the stability period improves by approximately 26%, 7%, 31%, and 150% compared to ESEP, P-SEP, Z-SEP, and LEACH, respectively. These results demonstrate that, in comparison with existing protocols, nodes in EZS-SEP deplete their energy more gradually. By incorporating both energy and distance factors into the CH selection process, our proposed protocol achieves a more balanced distribution of energy consumption, resulting in improved stability period of the network.

#### 5.3.3. Number of Dead nodes.

**Fig 9:**
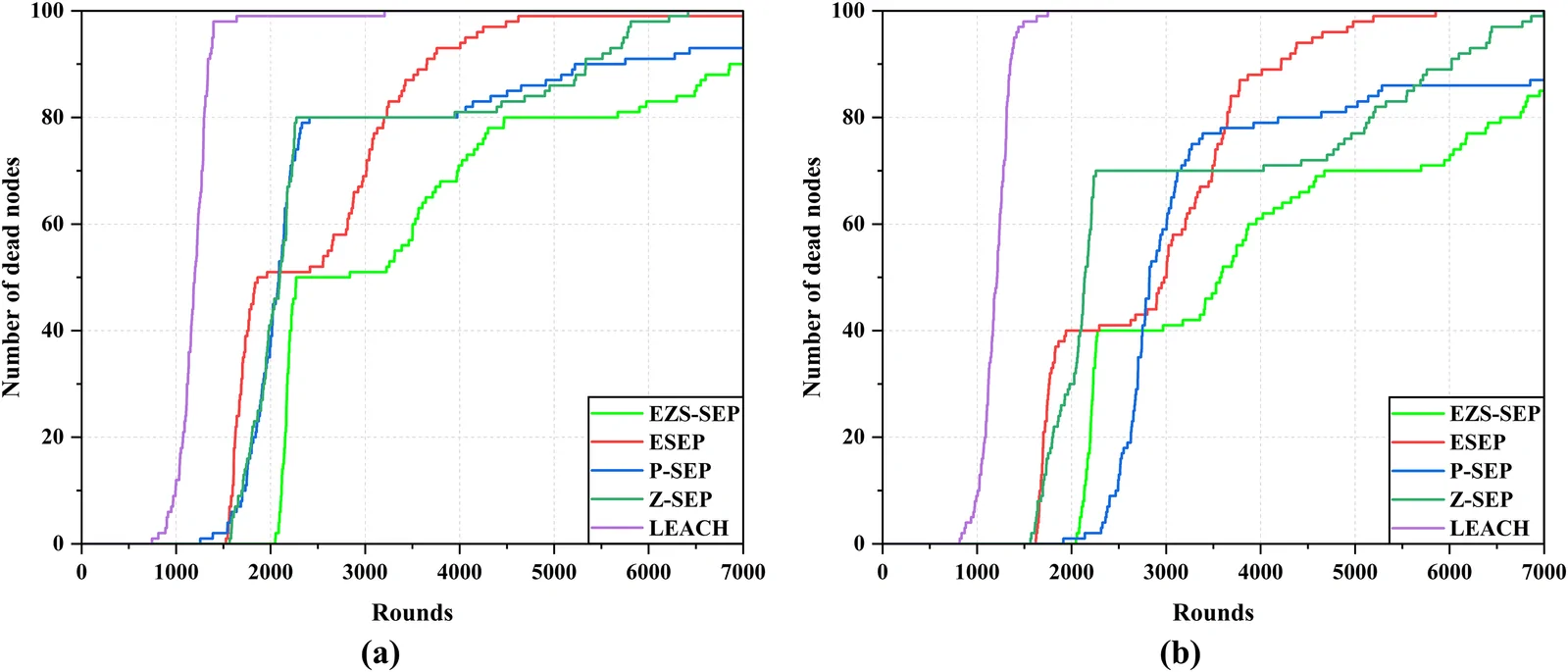
Dead nodes per round for (a) $m=0.2,m_{o}=0.3$ and (b) $m=0.3,m_{o}=0.3$.

**Fig 10:**
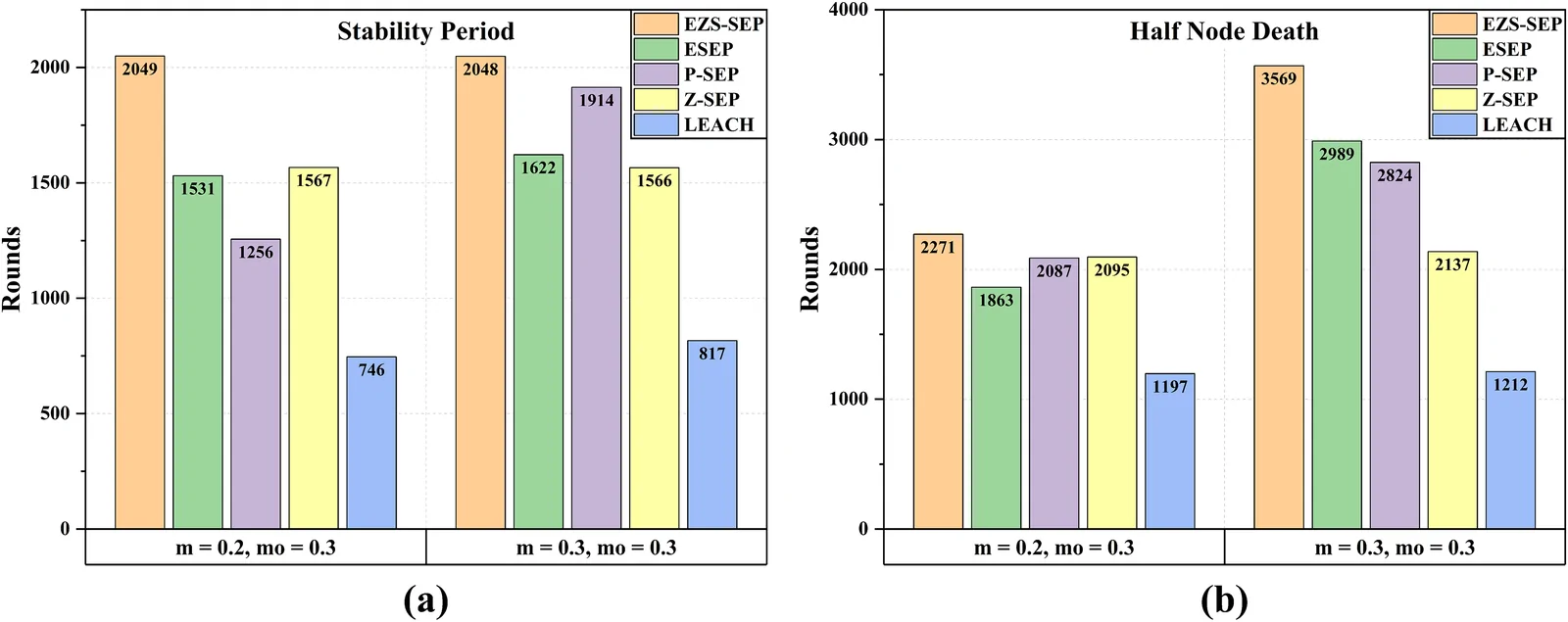
Comparison of (a) Stability Period (FND) and (b) Half Node Death (HND).

In Fig 9, the number of dead nodes in each round is compared across different protocols with our protocol under two conditions: (a) $m=\text{0}.\text{2},m_{o}=\text{0}.\text{3}$ and (b) $m=\text{0}.\text{3},m_{o}=\text{0}.\text{3}$. It can be observed that our protocol outperforms the others in terms of half-life. The HND values for condition (a) are 2271, 1863, 2087, 2095, and 1197 for EZS-SEP, ESEP, P-SEP, Z-SEP, and LEACH, respectively. This corresponds to improvements of approximately 22%, 9%, 8%, and 90% in half-node death compared to ESEP, P-SEP, Z-SEP, and LEACH. For condition (b), the HND values are 3569, 2989, 2824, 2137, and 1212 for EZS-SEP, ESEP, P-SEP, Z-SEP, and LEACH, respectively. These represent improvements of about 19%, 26%, 67%, and 194% in the network half lifetime compared to ESEP, P-SEP, Z-SEP, and LEACH. Therefore, the enhancements in EZS-SEP effectively prolong the network stability and facilitate optimal energy consumption among the nodes. Fig 10 visualizes the improvement of FND and HND between EZS-SEP and other existing protocols.

#### 5.3.4. Packets sent to BS.

**Fig 11:**
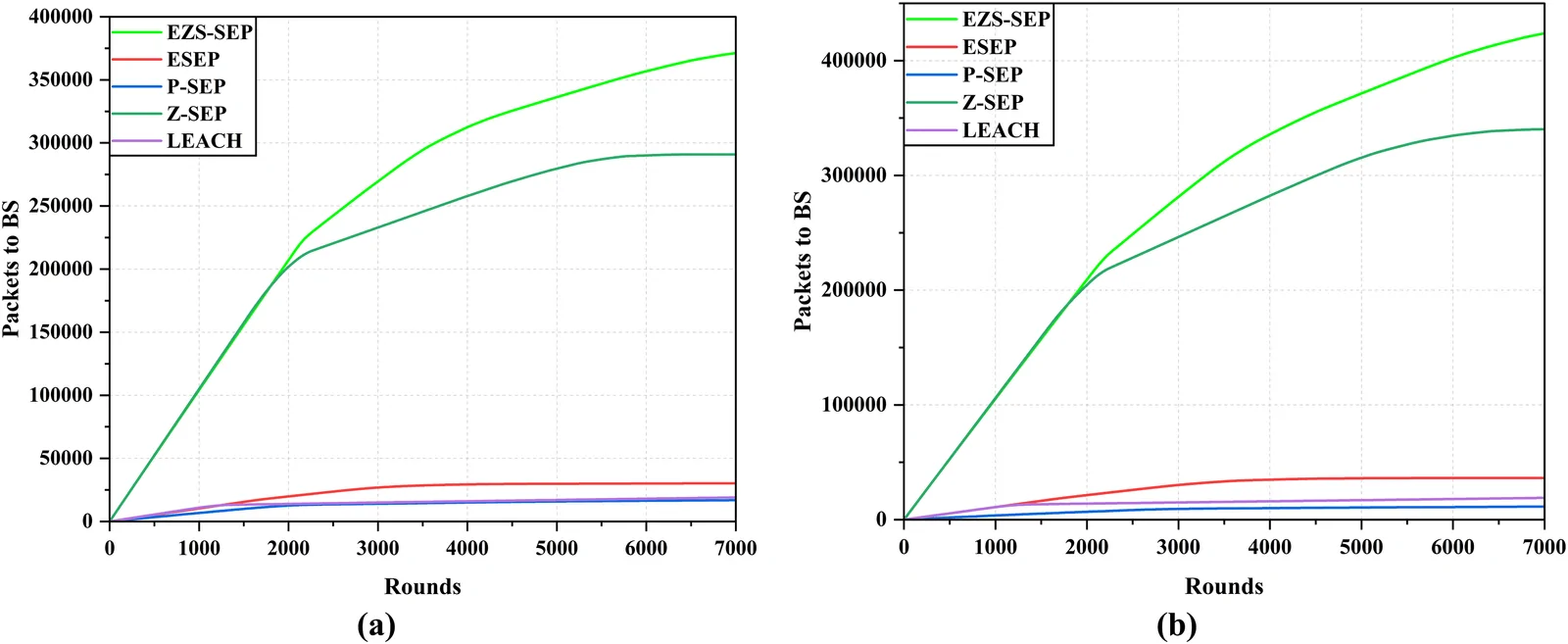
Packets to BS for (a) $m=0.2,m_{o}=0.3$ and (b) $m=0.3,m_{o}=0.3$.

We compared the number of packets sent to the BS under two different conditions between our protocol and several existing protocols. As EZS-SEP offers a longer network lifetime, a greater number of packets are sent to BS from the CHs. An increase in m and $m_{o}$ results in higher throughput, as more high-energy nodes are incorporated into the network. From Fig 11, significant differences in packets to the BS can be observed between our protocol and the others. For case (a), the number of packets is 371270, 30280, 16842, 290890, and 19047 for EZS-SEP, ESEP, P-SEP, Z-SEP, and LEACH, respectively. Only Z-SEP comes close to EZS-SEP, while the other protocols fall far behind the results achieved by our proposed protocol. For case (b), the scenario is similar, with packets to the BS being 423916, 36286, 11384, 340250, and 18999 for EZS-SEP, ESEP, P-SEP, Z-SEP, and LEACH, respectively. These results demonstrate a substantial improvement in this performance metric. The improvements of FND, HND and Throughput are tabulated in Table 5 for better understanding.

**Table 5:** Comparison of EZS-SEP with existing protocols.

Protocol	Stability Period		Half Lifetime		Throughput	
	m = 0.2 $m_{o}$ = 0.3	m = 0.3 $m_{o}$= 0.3	m = 0.2 $m_{o}$= 0.3	m = 0.3 $m_{o}$= 0.3	m = 0.2 $m_{o}$= 0.3	m = 0.3 $m_{o}$ 0.=3
EZS-SEP	**2049**	**2048**	**2271**	**3569**	**371270**	**423916**
ESEP	1531	1622	1863	2989	30280	36286
P-SEP	1256	1914	2087	2824	16842	11384
Z-SEP	1567	1566	2095	2137	290890	340250
LEACH	746	817	1197	1212	19047	18999

#### 5.3.5. Normalized Residual Energy of the Network.

**Fig 12:**
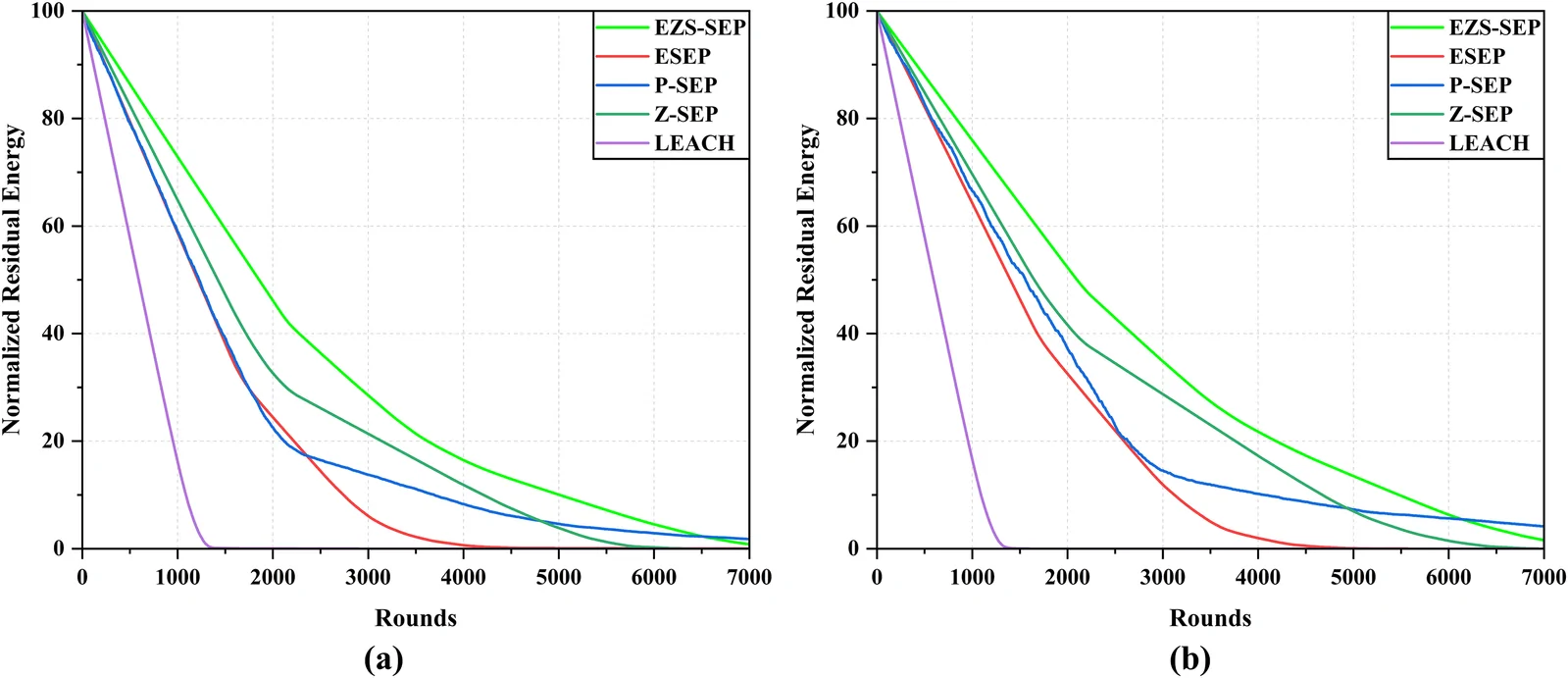
Normalized Residual Energy for (a) $m=0.2,m_{o}=0.3$ and (b) $m=0.3,m_{o}=0.3$.

Fig 12 represents the normalized residual energy or the total percentage remaining throughout the simulation. We can see that EZS-SEP uses less amount of energy during the whole simulation and less energy is consumed compared to ESEP, P-SEP, Z-SEP and LEACH respectively for both cases (a) $m=\text{0}.\text{2},m_{o}=\text{0}.\text{3}$ and (b) $m=\text{0}.\text{3},m_{o}=\text{0}.\text{3}$. For case (a), at 2000^th^ round, the normalized residual energy of EZS-SEP is 21.73%, 23.68%, 13.6% and 46.2% more than ESEP, P-SEP, Z-SEP and LEACH respectively. Similar results are obtained for second case (b), where the normalized residual energy is about 19.78%, 15.09%, 10.7% and 52.31% higher than ESEP, P-SEP, Z-SEP and LEACH respectively. This enhancement in performance is mainly due to considering optimum CH selection for the transmission of data. The process guarantees efficient energy utilization during data transmission by preserving the heterogeneity of the sensor network.

#### 5.3.6. Variation of Stability Period & Throughput.

We have observed the effect of varying number of the advanced and intermediate node on the stability period and throughput. By changing the fraction from 0.1 to 0.3 we have tabulated the data from the simulation. Table 6 shows the variation of FND and throughput for varying m and $m_{o}$. From the table, we can understand that the stability period remains very stable even if the quantity of advanced and intermediate nodes are changed. Whereas with the increase in advanced and intermediate nodes the throughput increases exponentially. This rapid growth in packets sent to BS is caused by the increasing number of advanced and intermediate nodes which take part in CH selection and send more data to the sink per round. Thus, our suggested protocol’s performance is extremely consistent with respect to throughput and stability.

**Table 6:** Variation of stability period & throughput.

		Stability Period			Throughput		
		m			m		
$m_{o}$		**0.1**	**0.2**	**0.3**	**0.1**	**0.2**	**0.3**
	**0.1**	1989	2018	2033	287131	332140	384158
	**0.2**	2055	2045	2026	309033	350544	406740
	**0.3**	2009	2058	2031	326097	382092	426753

### 5.4. Different network scenarios

For further investigation of EZS-SEP, three distinct network scenarios are taken into account and a comparison is conducted with ESEP, P-SEP, Z-SEP, and LEACH. Two sets of values are considered: (a) $m=\text{0}.\text{2},m_{o}=\text{0}.\text{3}$ and (b) $m=\text{0}.\text{3},m_{o}=\text{0}.\text{3}$ for both cases. The performance metrics compared include stability period, half node death, throughput, and remaining residual energy. The network scenarios considered are (i) changing the initial energy of nodes, (ii) changing the position of the sink and (iii) changing the network size.

#### 5.4.1. Varying the initial energy of nodes.

**Fig 13:**
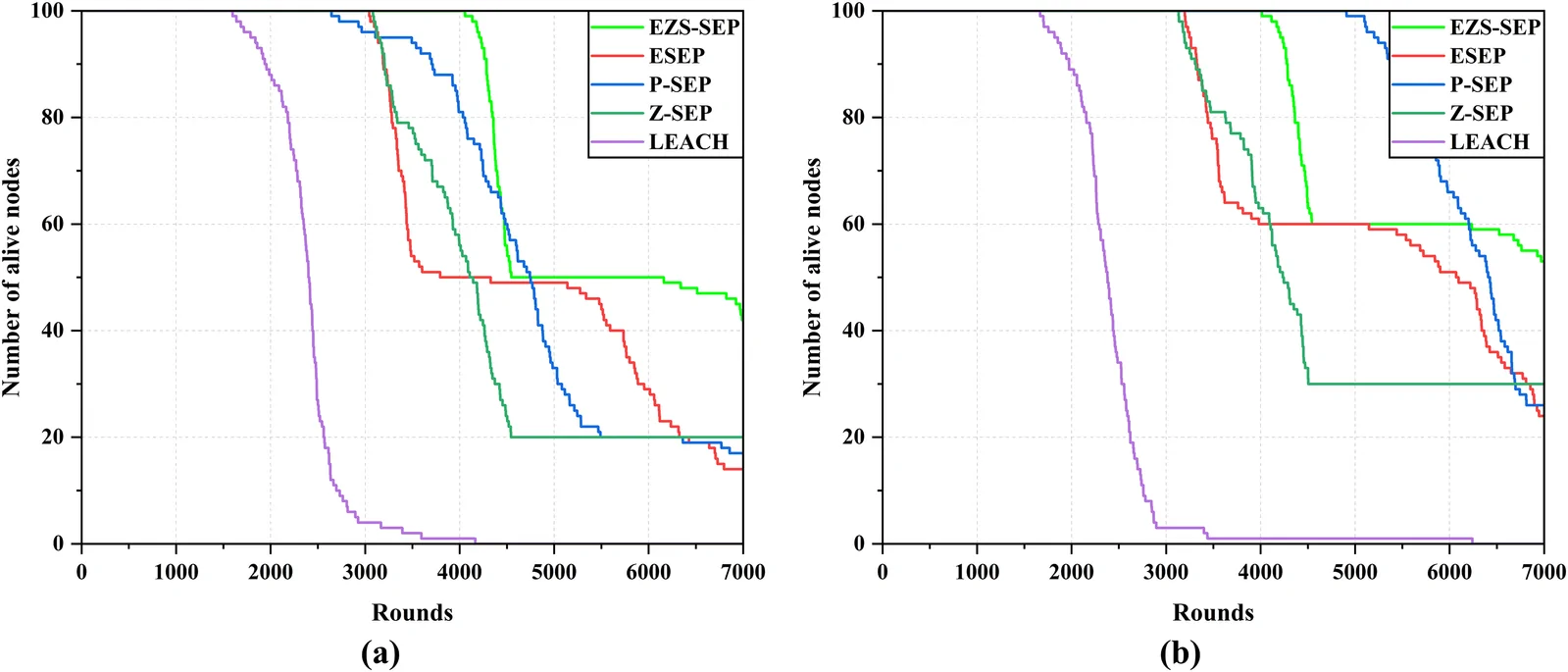
Alive nodes per round $(E_{o}=1j)$ for (a) $m=0.2,m_{o}=0.3$ and (b) $m=0.3,m_{o}=0.3$.

**Fig 14:**
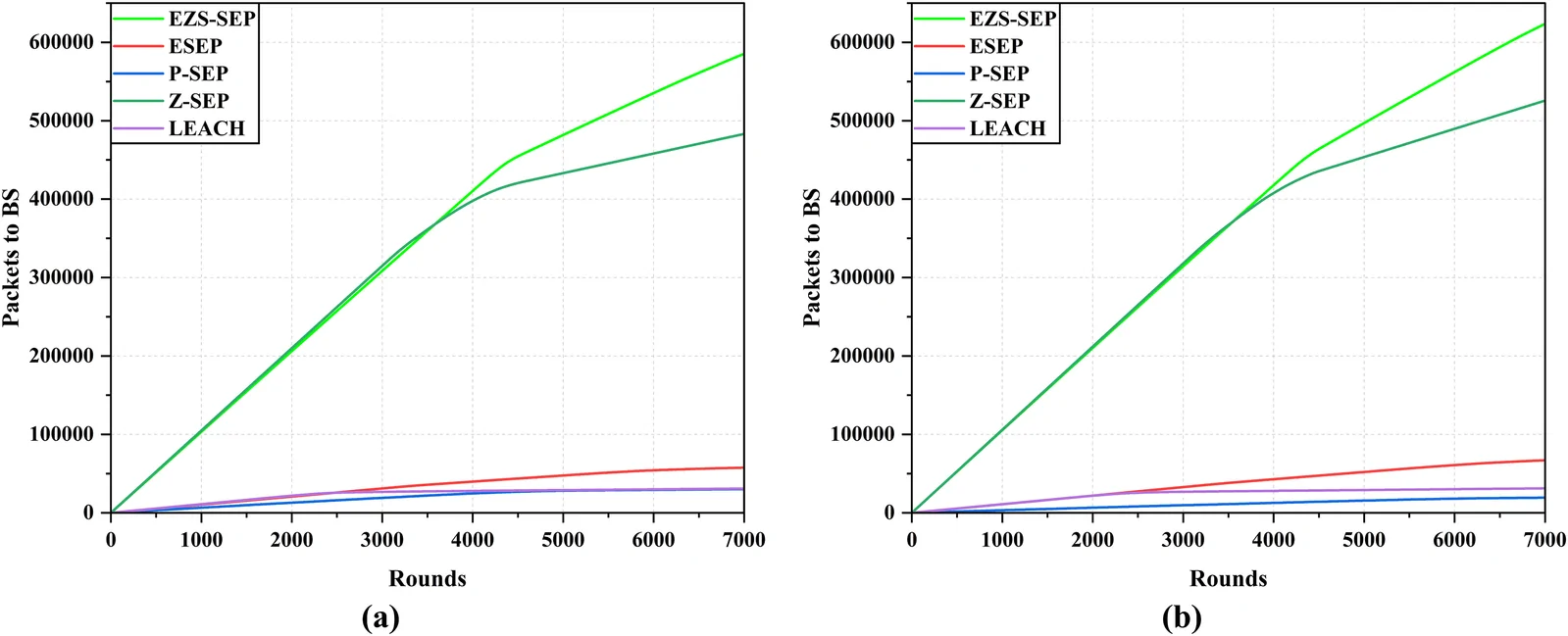
Packets to BS $(E_{o}=1j)$ for (a) $m=0.2,m_{o}=0.3$ and (b) $m=0.3,m_{o}=0.3$.

**Fig 15:**
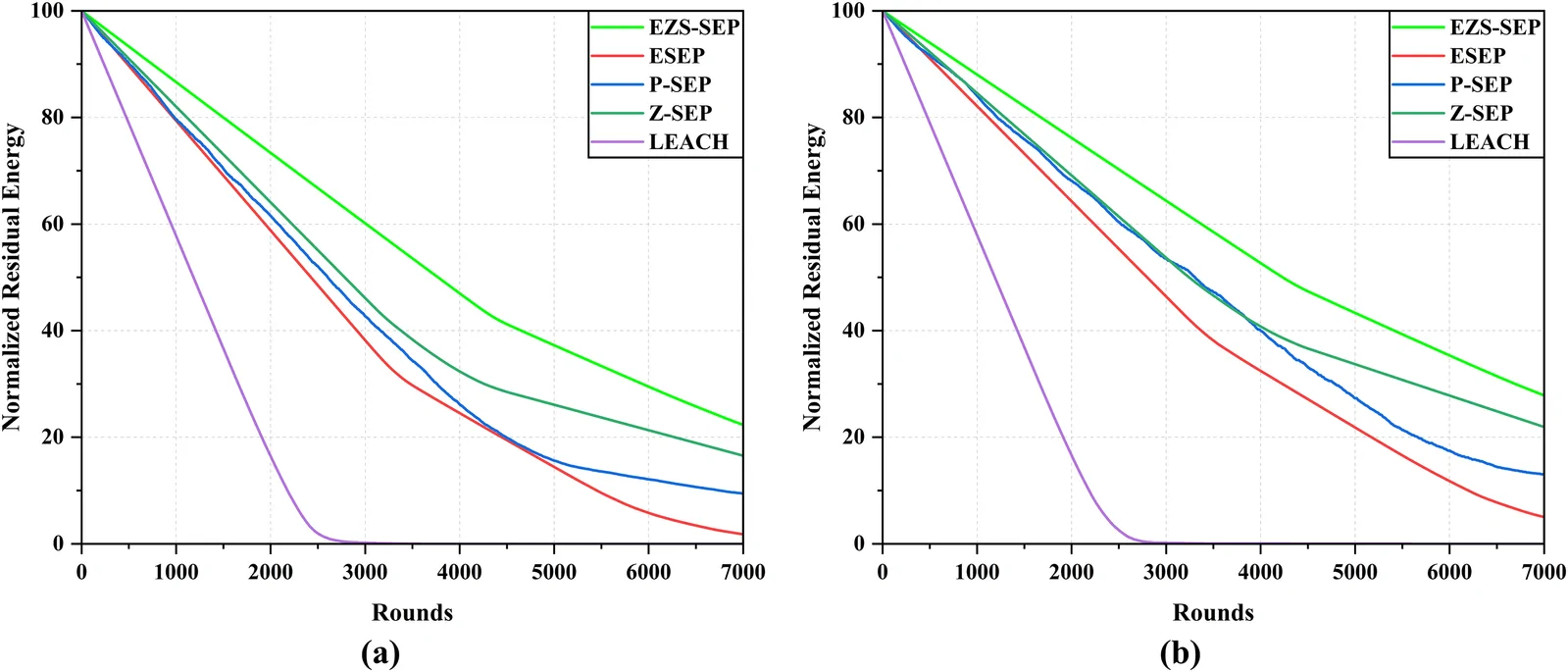
Normalized Residual Energy $(E_{o}=1j)$ for (a) $m=0.2,m_{o}=0.3$ and (b) $m=0.3,m_{o}=0.3$.

In WSN, initial energy of a node is a very crucial parameter for the efficiency of the network as the nodes are equipped with limited power. It affects the overall stability, throughput and energy usage of the whole network. Increasing the initial energy would result in an increased network lifetime as the nodes have more energy for data transmission for a long period of time. In this scenario, we have changed the value of the initial energy $\left(E_{o}\right)$ from 0.5 J to 1 J and compared the results with existing protocols. Fig 13–15 show the changed graphs for alive nodes per round, packets to BS and remaining energy percentage. We can see from Fig 13 that the stability period for EZS-SEP is 4013 and for ESEP, P-SEP, Z-SEP and LEACH the values are 2864, 2917, 3093 and 1678 respectively when m = 0.2; in case of m = 0.3 the values are 4015, 3198, 4909, 3133 and 1668 for EZS-SEP, ESEP, P-SEP, Z-SEP and LEACH sequentially. In case of HND, when m = 0.2 we can see that its 4550 for EZS-SEP and 3636, 4691, 4057 and 2393 for ESEP, P-SEP, Z-SEP and LEACH respectively. When m = 0.3 the value for EZS-SEP is 7659, whereas it is 6095, 6426, 4245 and 2382 for ESEP, P-SEP, Z-SEP and LEACH respectively. So, EZS-SEP expands the stability period in contrast to other protocols.

EZS-SEP transmits significantly more packets to the BS than other protocols as the lifetime increases significantly, as seen in Fig 14 when the initial energy is increased to 1 J. For m = 0.2 the packets sent to BS is 585311 and for m = 0.3 the number is 623677 in EZS-SEP, thus it sends much more packets to the BS than other protocols. Similar improvement is seen in case of remaining residual energy where more energy remains unused in case of EZS-SEP, which means less energy is consumed by EZS-SEP than the other protocols. Normalized residual energy is illustrated in Fig 15. We can see for m = 0.2, after 2000^th^ round the residual energy of EZS-SEP is 14.55%, 11.77%, 9.2% and 56.92% more than ESEP, P-SEP, Z-SEP and LEACH respectively. For m = 0.3, the remaining energy is 11.89%, 8.06%, 7.04% and 59.55% more after the 2000^th^ round.

#### 5.4.2. Changing the BS position.

**Fig 16:**
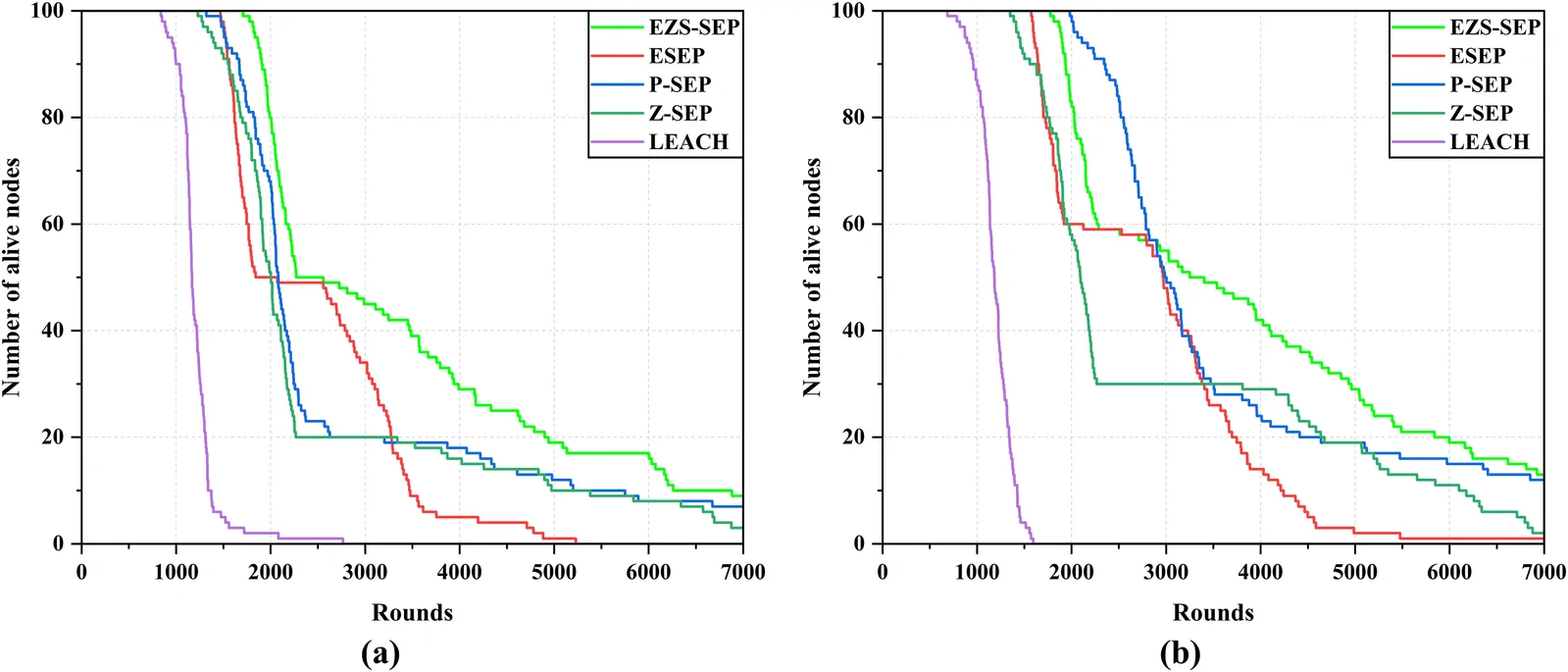
Alive nodes per round when BS at (35, 35) for (a) $m=0.2,m_{o}=0.3$ and (b) $m=0.3,m_{o}=0.3$.

In this instance, the position of the BS (X, Y) is moved to (35, 35) from (50, 50). When the BS is located at the center of the network, the average transmission distance between CHs and the BS is reduced, resulting in lower energy consumption during data transmission. Conversely, placing the BS at one side of the network increases the transmission distance for many nodes, leading to higher energy dissipation, a shorter stability period, and reduced throughput. The performance of EZS-SEP has been compared with the other protocols in this case by considering two values of m (0.2 and 0.3). This change causes significant variations in the results. For m = 0.2, the stability period of EZS-SEP has decreased to 1698 which is still longer than ESEP, P-SEP, Z-SEP and LEACH which have 1476, 1318, 1260 and 774 respectively. The numbers are 1776, 1574, 1982, 1351 and 687 respectively for EZS-SEP, ESEP, P-SEP, Z-SEP and LEACH for m = 0.3. The HND for EZS-SEP has also decreased to 2285 when m = 0.2, which is still longer than the other protocols. For m = 0.3, HND is decreased to 3403 for EZS-SEP, still higher than other protocols. Fig 16 represents the alive nodes per round for this case.

**Fig 17:**
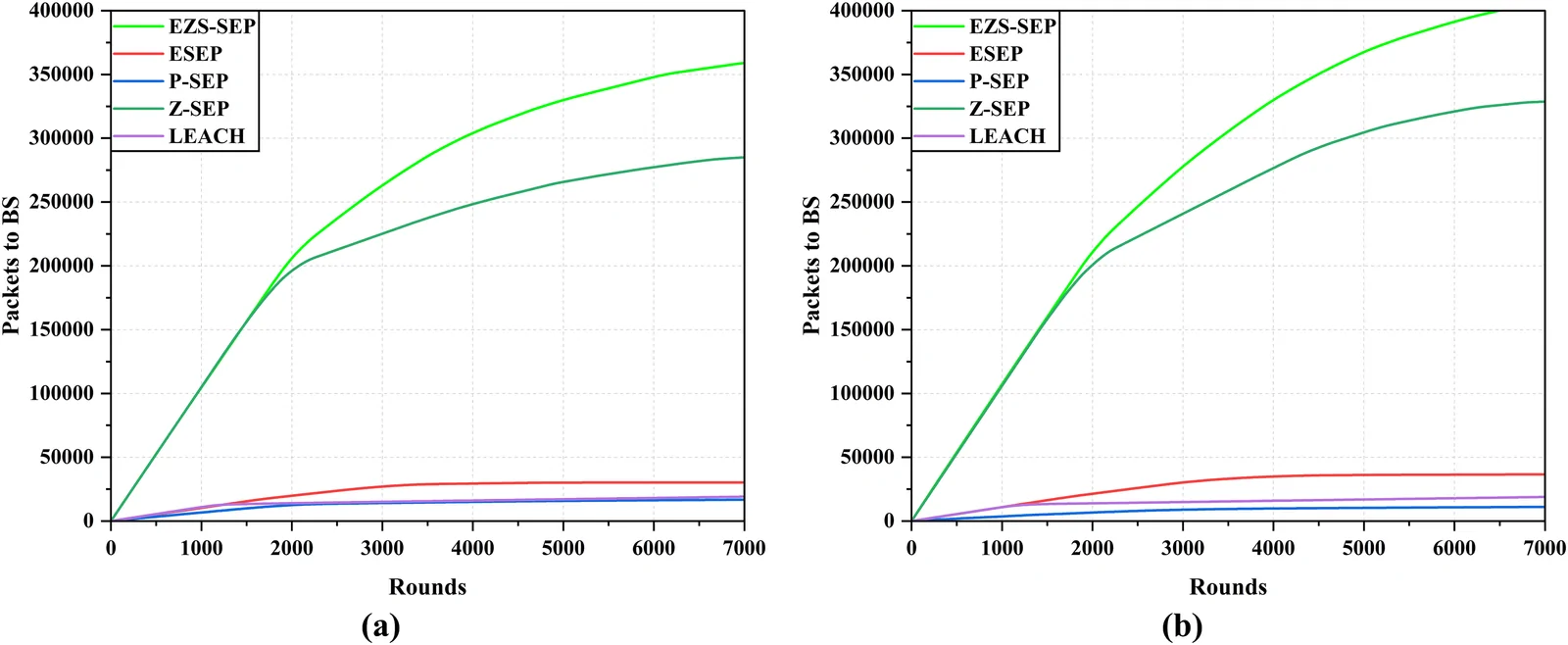
Packets to BS when BS at (35, 35) for (a) $m=0.2,m_{o}=0.3$ and (b) $m=0.3,m_{o}=0.3$.

**Fig 18:**
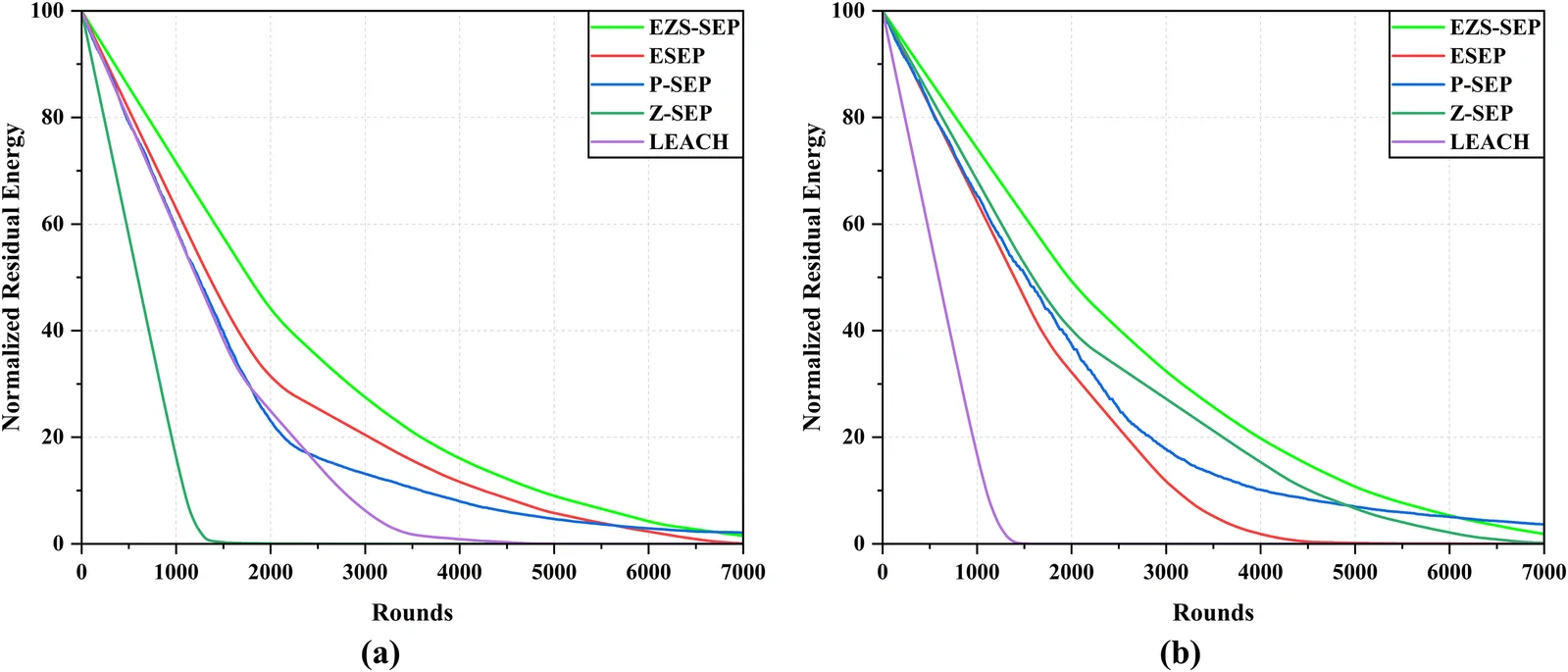
Normalized Residual Energy when BS at (35, 35) for (a) $m=0.2,m_{o}=0.3$ and (b) $m=0.3,m_{o}=0.3$.

Fig 17 shows that, compared with other protocols, EZS-SEP transmits more packets to the BS when BS is placed at (35, 35) for both m = 0.2 and m = 0.3. For both values of m the packets sent to BS are 366393 and 407651 in EZS-SEP which are much higher than existing protocols. Similar scenario is seen in case of normalized residual energy where less energy is consumed by EZS-SEP than the other protocols for both m = 0.2 and m = 0.3. Throughout the whole simulation we can see that less energy is consumed in our proposed protocol than the other protocol, which proves EZS-SEP is much more energy efficient. Fig 18 illustrates the normalized residual energy when BS is located at a different location.

#### 5.4.3. Changing the network size.

**Fig 19:**
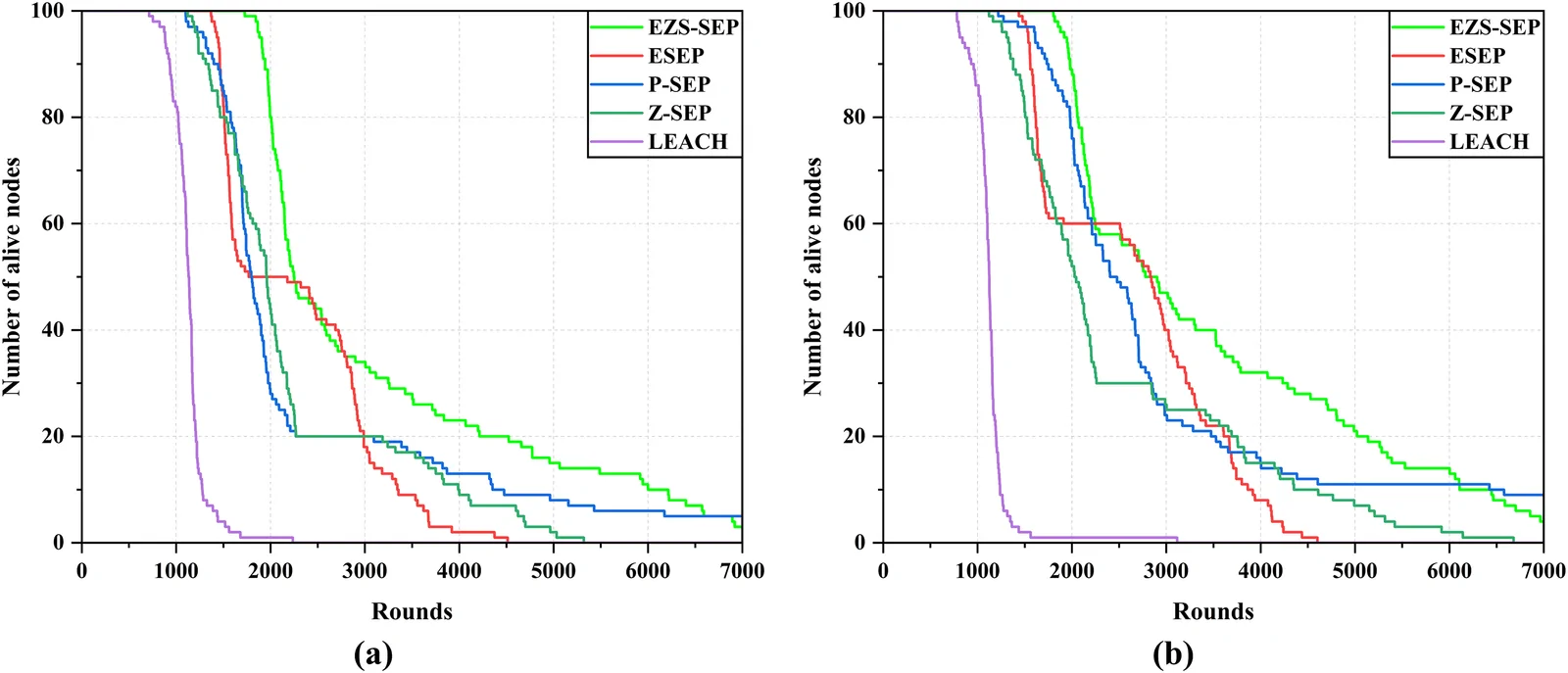
Alive nodes per round when Network Size (150, 150) for (a) $m=0.2,m_{o}=0.3$ and (b) $m=0.3,m_{o}=0.3$.

To demonstrate the scalability of the proposed protocol, the network size was increased from 100×100 m² to 150×150 m² while keeping the number of sensor nodes unchanged. Increasing the network dimensions enlarges the average communication distance between nodes, CHs, and the BS, leading to higher transmission energy consumption and a reduction in network lifetime. The performance of EZS-SEP under this larger deployment area was compared with ESEP, P-SEP, Z-SEP, and LEACH for m = 0.2 and m = 0.3. As expected, the increase in network size reduced the stability period of all protocols. However, EZS-SEP continued to achieve the longest stability period among the compared methods. For m = 0.2, the stability period of EZS-SEP was 1727 rounds, compared with 1369, 1101, 1123, and 714 rounds for ESEP, P-SEP, Z-SEP, and LEACH, respectively. Similarly, for m = 0.3, EZS-SEP achieved a stability period of 1804 rounds, whereas ESEP, P-SEP, Z-SEP, and LEACH attained 1439, 1219, 1121, and 781 rounds, respectively. These results demonstrate that the proposed protocol maintains better network stability even under a larger sensing field. The corresponding alive-node curves are illustrated in Fig 19.

**Fig 20:**
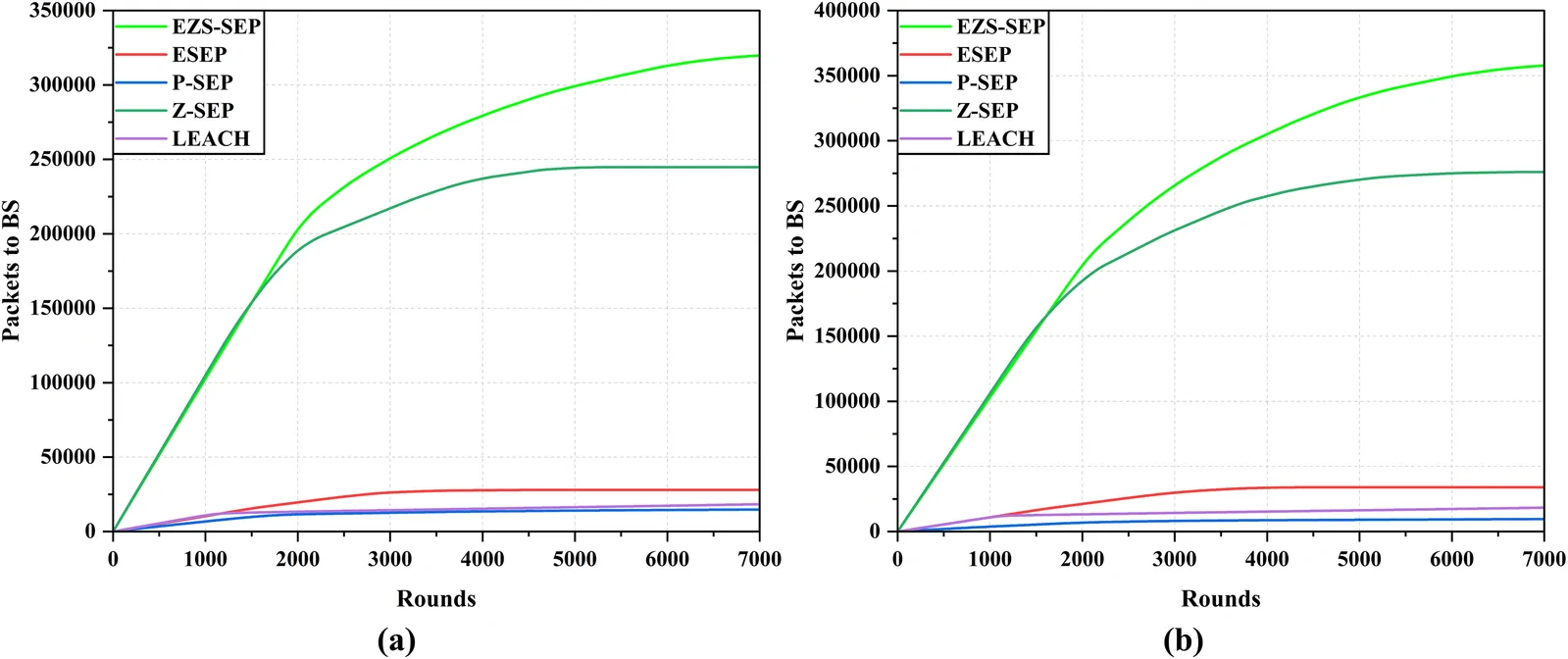
Packets to BS when Network Size (150, 150) for (a) $m=0.2,m_{o}=0.3$ and (b) $m=0.3,m_{o}=0.3$.

**Fig 21:**
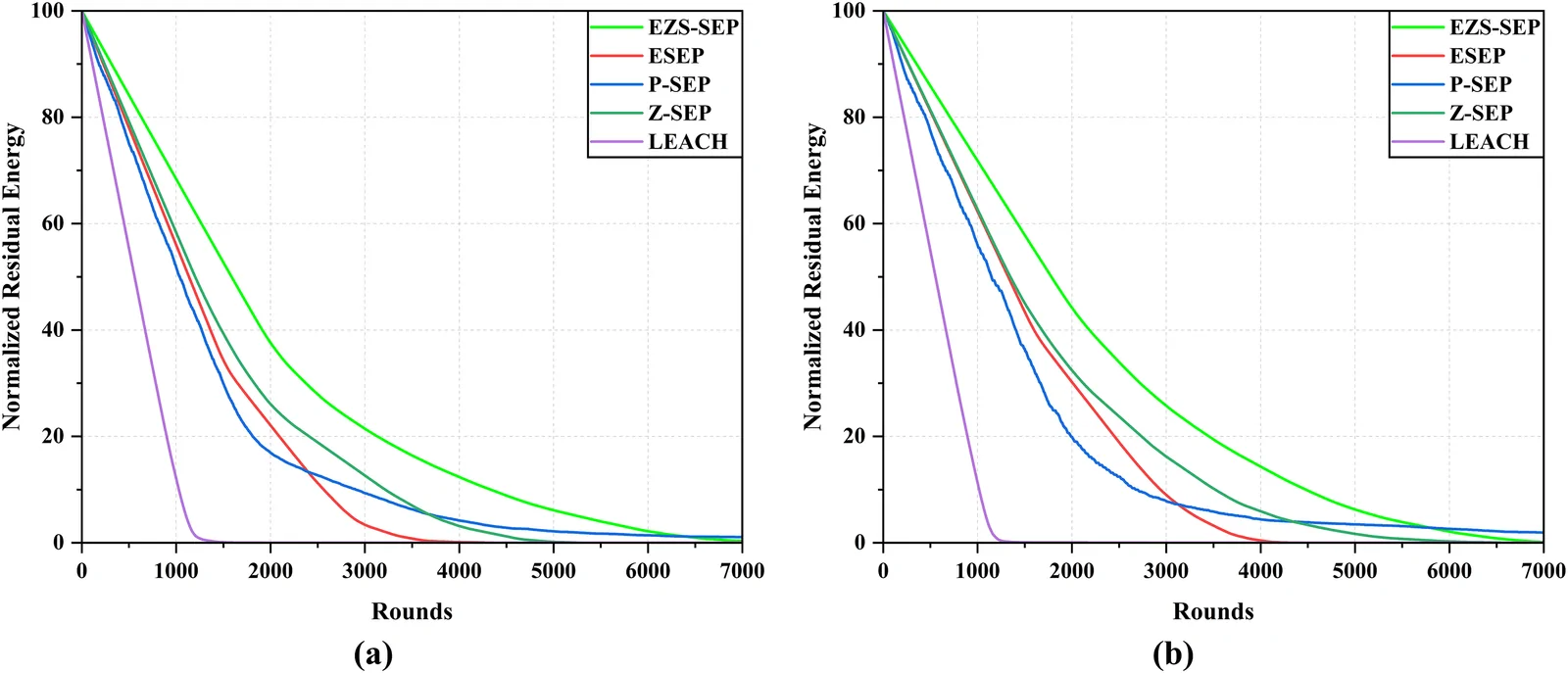
Normalized Residual Energy when Network Size (150, 150) for (a) $m=0.2,m_{o}=0.3$ and (b) $m=0.3,m_{o}=0.3$.

Fig 20 compares the packets transmitted to the BS for the enlarged network. Due to the increased communication distance, the throughput of all protocols decreases; however, EZS-SEP still achieves the highest throughput. At the 7000^th^ round, EZS-SEP delivers 319,764 packets for m = 0.2 and 357,749 packets for m = 0.3, outperforming ESEP, P-SEP, Z-SEP, and LEACH, demonstrating its superior data transmission capability. The normalized residual energy under the larger network is shown in Fig 21. EZS-SEP consistently retains more residual energy than the other protocols, indicating more balanced energy utilization. At the 2000^th^ round, EZS-SEP retains 37.60% and 44.22% of the initial energy for m = 0.2 and m = 0.3, respectively, which is higher than the competing protocols. These results confirm that EZS-SEP maintains better energy efficiency and throughput even as the network size increases.

From this extensive comparison, it can be concluded that EZS-SEP outperforms several existing protocols under varying network scenarios and parameters. The stability period is significantly extended, and high throughput is achieved along with efficient energy consumption. Table 7 presents the comparative analysis of EZS-SEP and other protocols under different network conditions.

**Table 7:** Comparison of EZS-SEP with existing protocols in different network conditions.

Protocol	Stability Period				HND				Packets to BS			
	$X_{BS}$= 50 $Y_{BS}$= 50 $E_{o}$= 0.5J M= 100^2^	$X_{BS}$= 50 $Y_{BS}$= 50 $E_{o}$= 1J M= 100^2^	$X_{BS}$= 35 $Y_{BS}$= 35 $E_{o}$= 0.5J M= 100^2^	$X_{BS}$= 50 $Y_{BS}$= 50 $E_{o}$= 0.5J M= 150^2^	$X_{BS}$= 50 $Y_{BS}$= 50 $E_{o}$= 0.5J M= 100^2^	$X_{BS}$= 50 $Y_{BS}$= 50 $E_{o}$= 1J M= 100^2^	$X_{BS}$= 35 $Y_{BS}$= 35 $E_{o}$= 0.5J M= 100^2^	$X_{BS}$= 50 $Y_{BS}$= 50 $E_{o}$= 0.5J M= 150^2^	$X_{BS}$= 50 $Y_{BS}$= 50 $E_{o}$= 0.5J M= 100^2^	$X_{BS}$= 50 $Y_{BS}$= 50 $E_{o}$= 1J M= 100^2^	$X_{BS}$= 35 $Y_{BS}$= 35 $E_{o}$= 0.5J M= 100^2^	$X_{BS}$= 50 $Y_{BS}$= 50 $E_{o}$= 0.5J M= 150^2^
EZS-SEP	**2049**	**4013**	**1698**	**1727**	**2271**	4550	**2285**	**2247**	**371270**	**687080**	**329609**	**319764**
ESEP	1531	2864	1476	1369	1863	3636	1876	1769	30280	60567	30509	27909
P-SEP	1256	2917	1318	1101	2087	**4691**	2086	1796	16842	33930	16945	14785
Z-SEP	1567	3093	1260	1123	2095	4057	1956	1957	290890	553691	260082	244789
LEACH	746	1678	774	714	1197	2393	1177	1132	19047	33934	16900	18356

### 5.5. Further Comparison with Recent State-of-the-art Routing Protocols

Table 8 provides a comparison between the proposed EZS-SEP and some of the recent state-of-the-art WSN routing protocols in terms of energy level, CH selection criteria, data transmission strategy, and network performance. Unlike the majority of the protocols, EZS-SEP takes into consideration the initial energy, the residual energy, the average network energy and the distance from the nodes to the BS during its clustering process making it more adaptive and energy balanced. Some protocols benefit from a multi-hop approach or from a metaheuristic optimization, but they typically increase the communication overhead and the complexity of computation. In contrast, EZS-SEP attains a high network lifetime (Last Node Death (LND) = 8081 rounds), competitive FND (2049 rounds) and HND (2271 rounds) and high throughput (3.87 × 10^5^ packets) among the compared methods while maintaining a simple hybrid communication mechanism, demonstrating its effectiveness in prolonging network stability and improving overall energy efficiency.

**Table 8:** Comparison of EZS-SEP with state-of-the-art routing protocols.

Ref.	Energy Level	CH Selection Parameters				Data Transmission	FND	HND	LND	TP
		Initial Energy	Residual Energy	Average Energy	Distance					
[13]	2	×	✓	✓	✓	Hybrid + Multi hop	670	1590	~8000	2×10^5^
[38]	3	✓	×	✓	✓	Single hop	1533	2338	~5500	1.86×10^4^
[53]	2	×	✓	✓	✓	Hybrid	1798	2215	3823	2.55×10^5^
[6]	Multi	✓	✓	✓	✓	Hybrid + Multi hop	870	1200	6683	1.46×10^5^
[49]	3	×	✓	✓	×	Single hop	1669	2918	7889	2.32×10^5^
[50]	3	×	✓	✓	✓	Single hop + Dual hop	1791	3574	8235	2.59×10^5^
[48]	3	✓	×	✓	✓	Single hop	1100	1940	~3200	4.3×10^5^
[44]	4	✓	×	×	×	Hybrid	2140	2233	7615	2.88×10^5^
[46]	1	×	✓	✓	✓	Multi hop	469	583	713	2×10^8^
[29]	1	×	✓	×	✓	Multi hop	2033	2666	2857	2.5×10^4^
EZS-SEP	3	✓	✓	✓	✓	Hybrid	2049	2271	8081	3.87×10^5^

### 5.6. Impact of key CH Selection Parameters

To investigate the contribution of each CH selection parameter, an ablation study was performed by removing one parameter at a time while keeping the others unchanged. The results in Table 9 show that EZS-SEP achieves the best overall performance when initial energy, residual energy, average network energy, and distance metrics are considered together. We have considered m = 0.2, $m_{o}$ = 0.3, α = 3 and β = 1.5 for this study.

**Table 9:** Impact of Key CH parameters.

Observations	CH Selection Parameters				FND	HND	LND	TP
	Initial Energy	Residual Energy	Average Energy	Distance				
1	×	✓	✓	✓	2005	2272	7431	3.7×10^5^
2	✓	×	✓	✓	2006	2272	7925	3.68×10^5^
3	✓	✓	×	✓	2006	**2273**	7530	3.71×10^5^
4	✓	✓	✓	×	1989	2227	7651	3.67×10^5^
5	✓	✓	✓	✓	**2049**	2271	**8081**	**3.87** × **10** ^**5**^

Removing the initial energy parameter produces the greatest reduction in network lifetime (7431), as nodes with higher initial energy are no longer prioritized for CH election during the early stages of network operation. Excluding the residual energy parameter reduces the throughput to 3.68×10^5^ packets, indicating less efficient energy utilization because nodes with low remaining energy may still be selected as CHs. Without considering the average network energy, the protocol achieves a lower lifetime (7530), reflecting poorer load balancing among nodes. Removing the distance parameter results in the lowest stability period (1989) and half lifetime (2227) because CHs farther from the BS require more transmission energy. Overall, integrating all four parameters enables EZS-SEP to select CHs that are both energy-rich and closer to the BS, resulting in the highest FND (2049), LND (8081), and throughput (3.87 × 10^5^ packets).

### 5.7. Statistical rigor and robustness analysis

**Fig 22:**
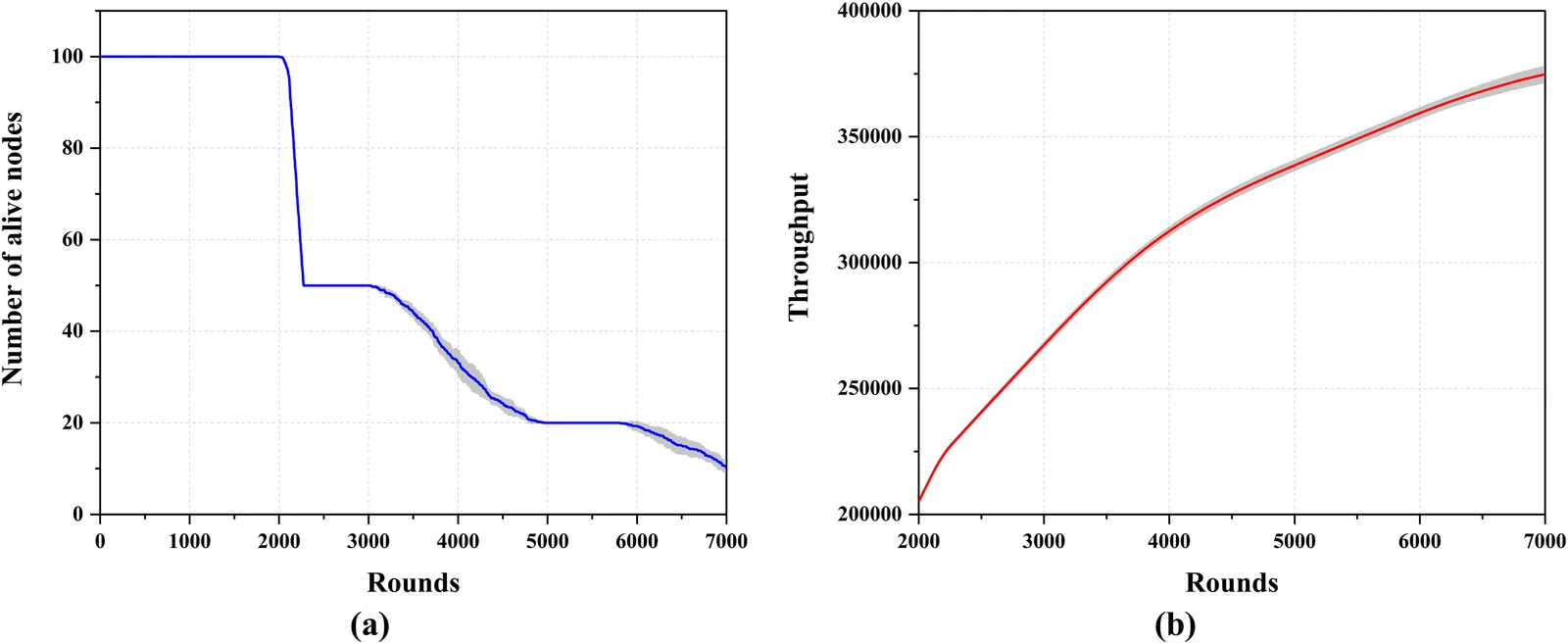
Performance evaluation of the proposed protocol over 10 random seeds: (a) Network lifetime and (b) Throughput.

To evaluate the robustness of the proposed protocol, each simulation scenario was executed using 10 independent random seeds. Fig 22 presents the mean performance with shaded regions representing the standard deviation. The per-round standard deviation of the number of alive nodes ranged from 0 to 3.87, with an average of 0.77, indicating very low variability across different runs. Most of the variation occurred during the rapid node depletion phase between 3000 and 5000 rounds and after 5900 rounds, while the mean stability period remained approximately 2047 rounds, demonstrating consistent network behavior. Similarly, the throughput exhibited minimal variation throughout both the growth and saturation phases, reaching an average of 374,746 packets after 7000 rounds. The standard deviation of the throughput ranged from 0 to 3519.58, with an average of 1623.99. The close agreement among the simulation runs confirms the robustness and reliability of EZS-SEP against random initialization. These results correspond to the configuration with $m=\text{0}.\text{2},m_{o}=\text{0}.\text{3}$ and α=3,β=1.5, further validating the consistency of the proposed protocol under this network setting.

### 5.8. Computational complexity analysis

The computational complexity of the proposed EZS-SEP is analyzed and compared with SEP and Z-SEP. Let N denote the total number of sensor nodes, K the number of elected CHs, R the number of communication rounds, and T the number of SOM training epochs. In SEP [17], nodes are randomly deployed with an initialization complexity of O(N). During each communication round, dead node detection, CH election, cluster formation, and energy updates are performed. The dominant operation is cluster formation, where each node searches for the nearest CH, resulting in a complexity of O(NK). Since the expected number of CHs is K=pN (where p is the CH election probability), the per-round computational complexity becomes $O(N^{\text{2}})$, leading to an overall complexity of $C_{SEP}=O(RN^{\text{2}})$. Similarly, Z-SEP [23] introduces zone-based deployment but retains the same clustering mechanism as SEP. Consequently, its initialization complexity remains O(N), while both the per-round and overall computational complexities are $O(N^{\text{2}})$ and $O(RN^{\text{2}})$, respectively.

Unlike SEP and Z-SEP, the proposed EZS-SEP employs SOM-based node deployment, introducing a one-time initialization complexity of O(NT). During each communication round, additional computations for average residual energy and average node-to-BS distance are performed to improve CH selection. However, these operations require only linear time, O(N), and do not affect the dominant complexity. As in SEP and Z-SEP, cluster formation remains the most computationally intensive operation with complexity O(NK), yielding a per-round complexity of $O(N^{\text{2}})$. Therefore, the overall computational complexity of EZS-SEP is:


CEZS−SEP=O(NT+RN2)

A comparison of the computational complexities of SEP, Z-SEP, and EZS-SEP is presented in Table 10. Although EZS-SEP incurs an additional one-time preprocessing cost due to SOM training, it preserves the same asymptotic routing complexity as the existing protocols during network operation. This additional initialization overhead enables more balanced node deployment and energy-aware CH selection, resulting in improved energy efficiency, enhanced network stability, and prolonged network lifetime without compromising scalability.

**Table 10:** Summarizing the computational complexity of the three protocols.

Protocol	Initialization Complexity	Per-Round Complexity	Overall Complexity
SEP	O(N)	$O(N^{\text{2}})$	$O(RN^{\text{2}})$
Z-SEP	O(N)	$O(N^{\text{2}})$	$O(RN^{\text{2}})$
EZS-SEP	O(NT)	$O(N^{\text{2}})$	$O(NT+RN^{\text{2}})$

## 6. Conclusion and future works

Efficient Zonal Stable Election Protocol with SOM-based node deployment (EZS-SEP) represents a significant advancement compared to traditional WSN protocols, addressing major issues related to energy consumption, network stability, and overall performance. The incorporation of SOM-based node deployment and clustering ensures efficient load distribution over non-uniformly distributed sensor nodes, thereby balancing energy consumption throughout the network. In contrast to conventional algorithms that rely on random or simple CH election methods, EZS-SEP considers residual energy, average network energy, and proximity to the BS for CH selection. This adaptive strategy selects energy-rich and well-positioned nodes as CHs, reducing energy consumption, balancing the network load, and extending network lifetime. Consequently, EZS-SEP outperforms classical protocols such as LEACH, P-SEP, Z-SEP, and ESEP in terms of stability period, throughput, and overall data transmission efficiency, enabling more reliable and energy-efficient communication with the BS. However, it is aimed at the static heterogeneous WSNs and does not cater to the dynamic network condition during its operation. It does not perform online learning for adaptive CH selection or routing decisions as in RL-based methods, limiting its applicability in highly dynamic or mobile environments. Moreover, the single-hop communication between the CHs and the BS can lead to low energy efficiency in large-scale deployment.

The proposed EZS-SEP protocol is appropriate for long-term energy-constrained WSN applications like precision agriculture, environmental monitoring, industrial automation, smart cities, and disaster management. It is suitable for reliable and sustainable sensing in real-world environments because of its heterogeneous deployment of nodes and the energy-aware CH selection, which increases network coverage, balances the energy usage, and prolongs the network lifetime.

In future work, implementation of this protocol with a mobile sink and mobile nodes, while incorporating multi-hop communication for large-scale WSNs can be considered. Machine learning techniques may also be integrated for intelligent CH and relay selection, and mobility-aware clustering with dynamic routing mechanisms can be investigated to adapt the framework for mobile wireless networks such as MANETs and VANETs.
